# Improving Environmental Health Literacy and Justice through Environmental Exposure Results Communication

**DOI:** 10.3390/ijerph13070690

**Published:** 2016-07-08

**Authors:** Monica D. Ramirez-Andreotta, Julia Green Brody, Nathan Lothrop, Miranda Loh, Paloma I. Beamer, Phil Brown

**Affiliations:** 1 Department of Soil, Water, and Environmental Science, University of Arizona, 1177 East 4th Street, Tucson, AZ 85721, USA; 2 Silent Spring Institute, 320 Nevada Street, Suite 302, Newton, MA 02460, USA; brody@silentspring.org; 3 Mel and Enid Zuckerman College of Public Health, University of Arizona 1295 N Martin Ave, Tucson, AZ 85724, USA; lothrop@email.arizona.edu (N.L.); mloh@email.arizona.edu (M.L.); pbeamer@email.arizona.edu (P.I.B.); 4 Institute of Occupational Medicine, Research Avenue North Riccarton, Currie EH14 4AP, UK; 5 Department of Sociology and Anthropology and Department of Health Sciences, Northeastern University, 360 Huntington Avenue, 310INV, Boston, MA 02115, USA; p.brown@neu.edu

**Keywords:** biomonitoring, exposure assessment, environmental health literacy, environmental justice, hazardous waste, contextual model of learning

## Abstract

Understanding the short- and long-term impacts of a biomonitoring and exposure project and reporting personal results back to study participants is critical for guiding future efforts, especially in the context of environmental justice. The purpose of this study was to evaluate learning outcomes from environmental communication efforts and whether environmental health literacy goals were met in an environmental justice community. We conducted 14 interviews with parents who had participated in the University of Arizona’s Metals Exposure Study in Homes and analyzed their responses using NVivo, a qualitative data management and analysis program. Key findings were that participants used the data to cope with their challenging circumstances, the majority of participants described changing their families’ household behaviors, and participants reported specific interventions to reduce family exposures. The strength of this study is that it provides insight into what people learn and gain from such results communication efforts, what participants want to know, and what type of additional information participants need to advance their environmental health literacy. This information can help improve future report back efforts and advance environmental health and justice.

## 1. Introduction

Human biomonitoring assesses the presence and concentration of a chemical in humans by measuring the parent chemical, its metabolite, or reaction product in human blood, urine, breast milk, saliva, breath, and hair [1]. Biomonitoring of environmental chemicals has been considered the “gold standard” for assessing people’s exposure to pollution [1] and has become integral to public health surveillance. Since 1999, the Center for Disease Control (CDC) has been tracking personal exposures in a representative sample of the US population and now, up to 265 environmental chemicals are monitored [2]. Biomonitoring is becoming a key strategy for providing a scientific basis for prevention via exposure reduction and motivating action [3], and has become an effective vehicle to provide much-needed data to advance environmental epidemiology, environmental health policy, and regulation [4]. Though biomonitoring has advanced our understanding of exposures, there are still some gaps associated with our understanding of and use of biomonitoring data, such as limited toxicological, epidemiologic, toxicokinetic, and pharmacokinetic data and the lack of health-based values for comparison [5]. These gaps are further complicated by translational science, risk communication and ethical challenges, as well as the socio-ecological context and the political-economic structures that exit within the communities in which these studies are conducted [3,6].

### Theoretical and Analytical Frameworks

In this section, we cover the following theoretical areas to set the stage for the research described here: environmental justice, environmental health literacy, and Contextual Model of Learning.

#### Environmental Justice

With roots in the civil rights movement, the environmental justice (EJ) movement emerged from local communities’ struggles with toxic contamination in the U.S. Advocacy and protests in Warren County, North Carolina, and other areas in the 1970s–1980s were pioneering efforts that elevated the environmental justice movement to the next level. Shortly after, definitive studies emerged demonstrating the link between minority status, low socioeconomic status, and community proximity to toxic landfills [7,8]. Since then, EJ scholars and activists have demonstrated that environmental inequality is closely associated with minority standings (e.g., [9,10]). The current literature on EJ comprises a wide range of quantitative studies consistently concluding that environmental risk burdens, known or potential, are distributed inequitably across racial/ethnic minorities and individuals with lower socioeconomic status [11]. Lack of privilege, limited political influence, and linguistic isolation also play a role in environmental injustice [12,13,14]. Class differences, health [15] and rural health [16] disparities, as well as information disparities [17] need to be considered in order to account for all racial groups, including whites [18]. For example, a rural health determinant has been proposed that suggests that there may be cultural and environmental factors exclusive to towns, regions, or economic types (e.g., farming, mining, manufacturing, or federal/state government dependent) that may affect health behavior and health [16]. To effectively address rural health disparities one must acknowledge the nexus and complex interactions between individuals, culture, and environment [19], which is also the case when working with EJ communities.

In this article, Dewey-Humboldt, Arizona is the EJ community that will be our focus. This is a predominately white, rural community situated between legacy mining waste and an abandoned smelter. In 2008, this site was added to the National Priorities List and Dewey-Humboldt, Arizona is now home to the Iron King Mine and Humboldt Smelter Superfund Site (details in Section 2.1). Just as observed in the Appalachian region with a legacy of exploitation from industries that establish and abandon hazardous facilities [20], rural mining communities in the southwest also have an exploitative past. These actions have created economically impaired regions that, in turn, limit political debate and activism around environmental quality in order to have or maintain economic development and employment [13,18,20]. Community members living in these areas, especially active mining sites, feel as though they have to choose between economic growth and environmental quality. This challenge is exacerbated when information and power imbalances exist between the affected community and government and industry stakeholders [17]. In Dewey-Humboldt, Arizona, community members distrust government agencies, and are consistently advocating for more information and updates from the U.S. Environmental Protection Agency (US EPA) regarding the Superfund site [21]. In parallel, the local water utility served water that was exceeding the federal drinking water standards for arsenic and nitrate. It took a minimum of four years for the public water utility to reach compliance (details in Section 3.2). Compounding the challenges associated with information disparity and limited political influence, are rural health disparities [16]. Rural culture is a social determinant of health that needs to be considered when assessing justice in communities. These socio-cultural contexts have created numerous sites across the world where EJ is not being achieved.

#### Environmental Health Literacy

Recently, much attention has been given to the emerging and evolving field of environmental health literacy (EHL), a concept that bridges shared theories from the fields of risk communication, environmental health science, behavioral science, evaluation, communications, public health, and the social sciences [22]. The Society for Public Health Education defines EHL as the ability to “integrate concepts from both environmental literacy and health literacy to develop the wide range of skills and competencies in order to seek out, comprehend, evaluate, and use environmental health information to make informed choices, reduce health risks, improve quality of life and protect the environment” [23]. At its most fundamental level, EHL involves “an understanding of the connection between environmental exposures and human health” [24]. The definition of EHL is expanding from a quiescent, unidirectional model (knowledge acquisition) towards an action-based model that provides individuals and communities with the tools and information to understand their risks and to exert control over the environmental exposures that may lead to adverse health outcomes [24,25]. Reporting bio- and environmental monitoring data back to participants in environmental public health projects has proven to be a successful way in which to raise EHL. The literature reveals that participants of biomonitoring studies with a report back effort: (1) generally want their results; (2) learn a great deal about environmental health when studies reported individual results along with comparative benchmarks and some sort of interpretive context; (3) understand results without undue alarm; (4) increase their understanding of the scientific method; (5) self-initiate new networking in resource-related issues; (6) began to consider possible exposure reduction strategies; and (7) leverage the report back results to inform government officials to be more stringent in their cleanup efforts [6,25,26,27,28]. Distinct frameworks have been used to evaluate the effectiveness of these reporting back efforts and they include clinical ethics and community-based participatory research. In order to further our understanding of report back efforts, we need to recognize that new participatory scientific practices have emerged [3]. We can pull from other disciplinary fields to frame and understand the learning outcomes of exposure studies coupled with an extensive report back process within the purview of EHL. These learning assessment and evaluation challenges provide fertile ground to develop and field-test report back models and layouts, as well as to see what type of learning and literacy is gained from such report back efforts.

#### Contextual Model of Learning

The Contextual Model of Learning [29] was originally proposed as a method to determine learning in free-choice settings like museums and science centers. It is “a device for organizing the complexities of learning within free-choice settings”, with a foundational understanding that learning is a complex phenomenon situated within a series of contexts over time: personal, sociocultural, and physical [29]. Recognizing that reporting back exposure data is carving out a new informal education setting (learning outside of school classrooms) and is stimulating free-choice learning (learning that is driven by the needs and interests of the learner rather than an external authority), it is appropriate to pull frameworks and methodologies from the discipline of informal science education [25]. The Contextual Model of Learning recognizes the strong influences on individual’s learning and actions in the short- and long-term, including: (1) prior knowledge, experience, motivations, expectations, and degree of choice and control over learning (personal); (2) cultural and social relationships, cultural values and the interactions with others in their own social group as well as interactions with educators outside their common social group (sociocultural); and (3) placed-based experiences and one’s interface with the environment (physical). Communities neighboring contaminated sites are learning on their own about pollutants at hazardous waste sites and the associated health effects [25], and this type of learning is an iterative process that is place-based and socioculturally mediated [29,30]. A multi-faceted framework is needed when confronting multifactorial challenges that arise in EJ communities. Thus, it is appropriate to use a framework that recognizes the interplay and complexity in an EJ free-choice learning setting. The Contextual Model of Learning is important in community-based EJ efforts because it can capture how personal, sociocultural, and physical experiences influence learning and action. Understanding how people learn and their motivations can inform local-based EHL efforts, stimulate new forms of knowledge, and trigger more participation in environmental decision-making.

The objective of this study was to evaluate the experience of participants in the University of Arizona’s Metals Exposure Study in Homes (MESH), and to document participants’ understanding of results for their home environment and children, how they used the data, and any actions they had considered in response to their results. MESH is one of eight biomonitoring studies selected as part of the Personal Exposure Report-Back Ethics Study (PERE), a larger project that is: (1) examining how researchers report back data, Institutional Review Boards evaluate such protocols, and how participants understand and use results; and (2) using this information to develop best practices for results communication. Within the above frameworks, we evaluate learning outcomes from report back efforts and whether EHL goals are being met in an EJ community. Specifically, we aim to assess the following EHL issues put forth by Finn and O’Fallon (2015): (1) the relation between EHL and resilience, e.g., if it would increase an individual or community ability to cope in challenging circumstances; (2) whether messaging about environmental factors actually leads to behavioral changes; (3) whether messaging leads to prevention, reduction, or mitigation of environmental risk factors; and (4) different approaches for measuring success. We hypothesize that such documentation of potential learning and action using a free-choice learning framework would be an effective method by which to assess the report back efforts in EJ communities and, in effect, evaluate individual’s EHL related to metals in the environment.

## 2. Materials and Methods

### 2.1. Study Population

Sandwiched in between a 153-acre site of legacy mine tailings waste with arsenic concentrations exceeding 3700 mg/kg and a legacy smelter area occupying 189 acres is the Town of Dewey-Humboldt, Arizona, with a population of 3894 people [31]. The Iron King Mine and Humboldt Smelter Superfund site (Iron King) has ~4 million cubic meters of mine tailings containing arsenic and other contaminants, and is subject to wind and water erosion into adjacent locations [32,33,34]. In addition to the site, Dewey-Humboldt is located in Yavapai County, an area known for naturally high levels of arsenic in groundwater and soil due to the presence of granite bedrock and the arsenic-rich Supai Sandstone formation [35]. The Arizona Department of Environmental Quality (ADEQ) observed that nineteen percent of ground water samples (276 out of 1477 collected between 1995 and 2009) were above the arsenic drinking water standard of 10 micrograms per liter (μg/L), and the majority of these sites are located in Yavapai County where there have been concentrations in excess of 2000 μg/L [36]. This issue is further complicated by: (1) “small” (serving <3300 people) or “very small” (serving <500 people) water utilities struggling to meet federal health-based drinking water standards because of lack of technical and financial resources; and (2) private wells are not overseen by regulatory agencies and are the sole responsibility of the private well owner to determine their water quality. Lack of regulatory oversight and reliance on private wells is another form of environmental injustice due to the rural location. Furthermore, Yavapai County is a rural community and is considered a medically underserved population due to the low ratio of primary medical care physicians per 1000 individuals, infant mortality rates, percentage of the population with incomes below the poverty level, and percentage of the population age 65 or over [37].

### 2.2. The University of Arizona’s Metals Exposure Study in Homes

**Figure 1:**
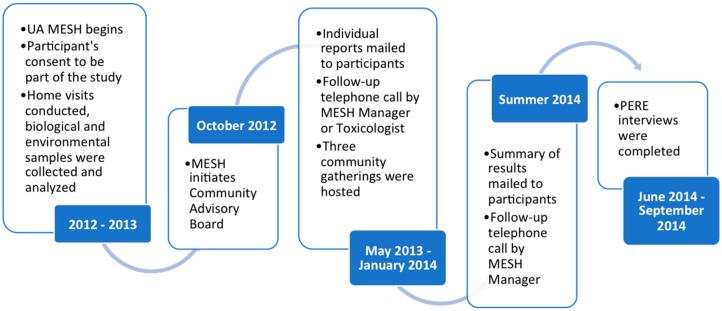
Study timeline.

**Figure 2:**
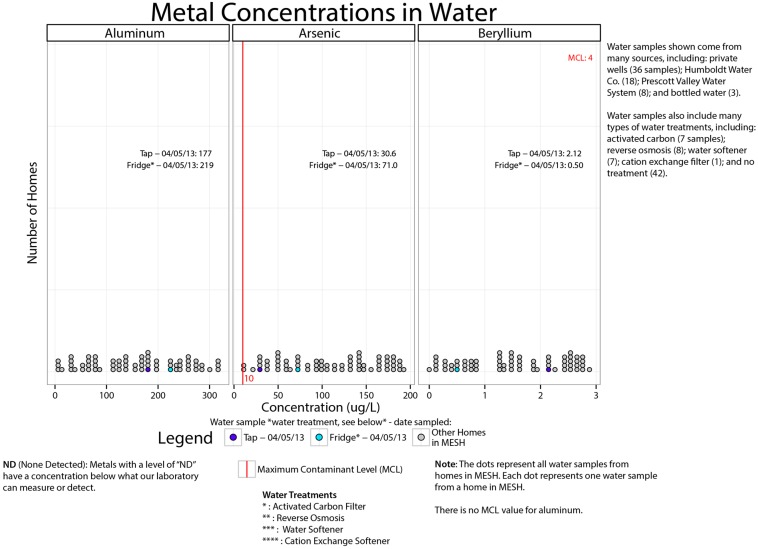
Example of a page in the summary of results packet mailed to MESH participants in summer 2014. Each graph shows the household’s data point(s) highlighted for comparison to other participating households and the MCL.

Beginning in 2008, members of the Dewey-Humboldt community partnered with the University of Arizona (UAZ) [26,38] on community-engaged environmental health research initiatives to characterize the extent of anthropogenic and naturally-occurring arsenic and heavy metal contamination in their residential areas. The MESH project, supported by the National Institute of Environmental Health Sciences’ Superfund Research Program at the University of Arizona (UA SRP), was developed in response to community concerns about exposure to metal(loid)s, such as arsenic, lead, cadmium, nickel, beryllium, aluminum, and chromium [34]. MESH assessed arsenic and heavy metal pathways and levels of exposure in local residents, specifically in local children 1–11 years of age. In 2012–2013, two home visits were conducted and biological (urine, toenails, and blood lead) and environmental samples (soil, dust, water) were collected and analyzed for metal(loid) content. Questionnaires were administered to ascertain children’s health and household activities and characteristics that might influence the levels of metals in the home environment. Parents also completed activity duration and dietary logs for the four days prior to the second home visit when environmental sampling occurred. To inform the study design, MESH researchers worked with pediatricians and the AZ Department of Health Services throughout the study. A total of 70 children, 1–11 years of age, from 34 homes in the Dewey-Humboldt area, participated in MESH [34]. As the blood samples were taken, participants were given individual blood lead results 2–3 weeks after testing (laboratory usually took ~1–2 weeks) via a “low blood lead” letter developed in consultation with a pediatrician who was part of the MESH team. An “over CDC action limit” letter was also generated, but never used since all children were below the CDC action limit of 5 micrograms per deciliter [39]. The MESH team reported the individual results (Supplemental Materials) in batches, which were sent out in May 2013 (half), October 2013 (one-forth), and the remaining in January 2014 (see Figure 1 for study timeline). During the summer of 2014 participants then received a follow-up in the form of a summary of results (aggregate with their data point(s) highlighted for comparison, see Figure 2 or Supplemental Materials for full summary of results report), as well as guidelines for best practices for limiting exposure (Table 1). Participants were contacted at least two weeks after receiving both their packets for follow-up. A MESH researcher (in this case, a toxicologist) would immediately contact a participant if any of the following occurred:

- The concentration of arsenic in their water sample was above 50 μg/L
- The concentration of lead in their water sample was above 15 μg/L (the current maximum contaminant level, MCL)
- Multiple children in home had creatinine-corrected urinary arsenic values over the 2013 National Health and Nutrition Examination Survey (NHANES) 95th percentile for children 6–11 years of age.

If a participant’s sample met any of the criteria above, the MESH team would first notify the participant by phone and then re-analyze the sample as soon as possible (which could be anywhere from a few days to a few weeks). If the concentration of arsenic or any other metal in water exceeded their respective MCL, the MESH manager would call participants. The MESH team considered calling participants if the concentration of arsenic or other metals in their soil or dust exceeded the ADEQ soil remediation levels (SRL), but since ADEQ SRLs are guidelines suggesting further investigation and are standards or action levels, the MESH team declined this idea.

In addition, starting in October 2012, the MESH team initiated a Community Advisory Board (CAB) and hosted seven meetings in October 2012 through June 2014. The CAB consisted of the former and current town mayor, a local congressional representative, community representatives, the UAZ Yavapai County Cooperative Extension Director, former MESH field technicians, and the UAZ Yavapai County Assistant Director of Public Health Services. Attendance varied by meeting. In November 2013, the MESH team hosted a research participants meeting to get feedback regarding the summary of results draft and to answer any questions they had about the results or study. Then, in January of 2014, the MESH team showed the CAB drafts of the summary of results packet and solicited additional feedback. Changes suggested by the CAB, such as increasing the number of infographics and reducing text, were made prior to finalizing and distributing the summary report.

### 2.3. Personal Exposure Report-Back Ethics Study

The PERE Study is an NIEHS-funded project that examines how diverse biomonitoring projects report results to participants and how those participants understand and use the information. At the beginning of the PERE Study, reporting individual exposure results was uncommon, so the PERE Study began by contacting principal investigators (PIs) in the few studies that had developed novel individual report-back methods. The PERE Study sought to include examples of academic, government, and citizen-led studies and later added other studies as new opportunities arose. Since participation required considerable support from each PI, studies were selected based on collegial and research relationships, and the PERE Study reached out to PIs of biomonitoring projects who were interested in the project goals. The rationale was that this selection method would increase the likelihood of accessing participants, researchers, and IRBs.

A member of the PERE Study research team worked with the MESH group to set up the participant interviews. First, the PERE research team member submitted a “Request for University of Arizona Human Research Site Authorization” along with the phone script, letter to MESH participants, and postcard to be used when re-contacting participants. Once approved, the MESH Project manager contacted (phone and mail) all MESH participants who had agreed to be re-contacted in the original informed consent. A total of 17 participants were re-contacted and 15 agreed to be interviewed by a member of the PERE research team. A member of the PERE study then contacted MESH participants by phone to schedule the interview about their experiences with the study and the report-back process. Fourteen interviews (41.2%) were conducted with 17 individuals (three interviews had two parents) between June and September 2014. During this semi-structured interview, participants were asked to describe their experience participating in the sampling, their understanding of results for their home, their emotional response, and any actions they had considered in response to their results. Each interview lasted between 36 and 100 min, and the median interview length was 68 min. For MESH, we interviewed participants and researchers, the UAZ IRB declined an interview. Only interview data from the MESH participants are reported here.

### 2.4. Analysis

Interviews were transcribed, and transcripts were imported into NVivo for Mac Version 10.1.3 (QSR International, Melbourne, Australia), a qualitative data management and analysis program. A coding scheme was created that closely resembled the PERE interview questionnaire. A member of the PERE research team conducted an initial analysis of the interviews and created a set of initial observations and preliminary response categories for each question. Response categories were adapted to better fit the interview material. In some cases, this involved creating new categories and merging categories. In addition to the systematic NVivo coding process, this research member constructed interview summaries for each participant describing overall impressions and detailing themes of action, EHL, report back preference, and general feedback. Then, the team member conducted a more detailed reading of each interview and coded each response into the appropriate category.

Comparisons (i.e., demographic parameters and children’s exposure levels) between those who were included in PERE versus those not included were examined using non-parametric Fisher’s Exact Test in STATA (version 13.1, StataCorp, College Station, TX, USA).

## 3. Results

**Figure 3:**
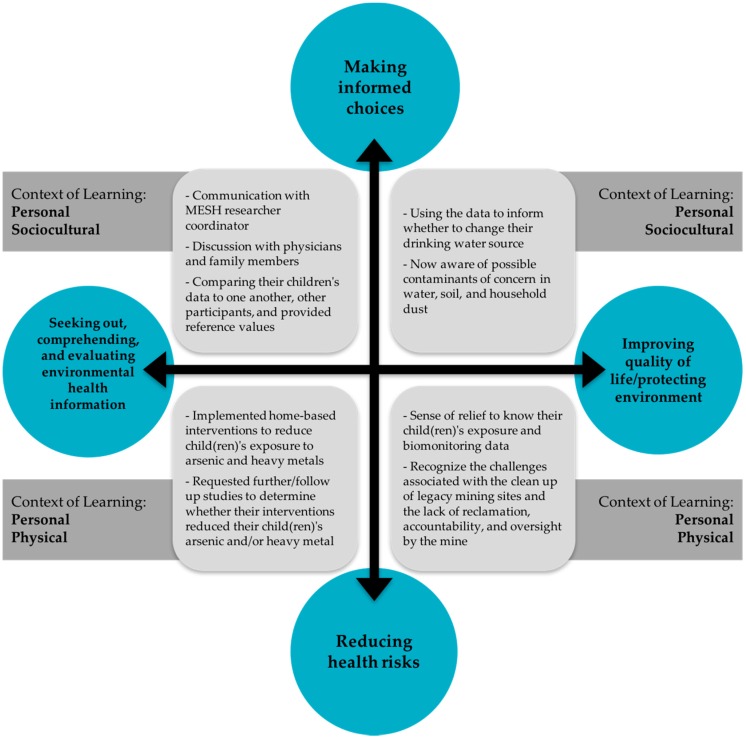
Participant’s context of learning, actions, and their alignment with environmental health literacy goals.

To determine the outcomes of reporting data back to participants and how it advanced their EHL, we analyzed the data in terms of: (1) seeking out, comprehending, and evaluating environmental health information; (2) using environmental health information to make informed choices and reduce health risks; and (3) improving quality of life and protecting the environment (Figure 3). Participants reported actions and interventions to reduce their child’s exposure (see Table 2), suggested news ways in which to communicate results to participants, and asked new research questions. Challenges that the MESH participants described were related to desired opportunities to network with one another, preference for additional information related to the metals of concern, requests for the results communication materials to include a geographic representation of the data, and more opportunities to interact with the MESH research team.

There were no significant differences in annual income, educational attainment, race/ethnicity, or number of households below the poverty level between those interviewed and included in PERE and not included (Table 3). When comparing children’s urinary inorganic-related arsenic concentrations (species and metabolites) [34,40] within MESH and then to the NHANES 2011–2012, there were no significant differences between those included in PERE versus those not included (Table 4). However, the lack of significant differences could be due to the limited sample size.

### 3.1. Why Parents Participated in MESH

All parents interviewed (*n* = 14) decided to participate in the study to ensure the safety of their children. Parents specifically stated that their children play outside and drink the water, and they wanted to see if their child(ren) had been exposed to arsenic and heavy metals. Eight participants specifically referred to wanting to get their water checked, and referenced past announcements about the public water quality (detailed in Section 3.2). One participant stressed wanting to protect her child and also stressed wanting to be part of the scientific investigation:

Because they’ve [US EPA] done air studies, and the air studies are saying … there’s not a lot of arsenic or lead in the particles in the dust, but somehow or another, I didn’t believe it; I really didn’t. … I’ve always wanted to see for myself what’s going on, and so that’s one of the reasons why I wanted to participate”.

In two households, it was the grandparents who enrolled their grandchildren in the study and two parents reported participating in the study because their mother encouraged them to enroll. In one instance, the grandparents witnessed the negative health effects of lead exposure, and in the other, the grandmother was worried about the water impacting the young children in the home. The role of the grandparent is highlighted here because to the best of our knowledge, this is the first time the role of an elder was brought up as a motivation for participating in a biomonitoring study. Additionally, nearly one million children in the US are living in homes where the grandparent is the householder and neither parent is present in the home [41,42]. These “grandfamilies” experience a unique set of challenges related to legal issues (adoption, legal custody or guardianship) and the child’s physical and mental health challenges due to the difficult situation that caused them to be placed in the grandparent’s care. Grandparent caregivers struggle to: obtain health insurance and supportive services for the child, care for a child on a fixed income, and maintain their own health [41]. Due to these extenuating circumstances, a nonparent care giver and child will most likely need to receive additional assistance to address environmental health issues and concerns.

### 3.2. Reducing Health Risks and Making Informed Choices

When participants were asked: “Have you considered making any changes to reduce chemical exposures as a result of the study?”, thirteen participants gave examples of actions that they had taken to reduce their exposure, and two more stated an action they plan to do. We anticipate that these actions occurred after participants received their individual household results since the summary booklet arrived between one to three weeks prior to the interviews and some of the activities would have required additional planning and time. It is important to note that within their individual household result booklets, web links to electronic information were provided for additional resources, whereas the summary booklet included two (hard copy) informational brochures (as mentioned in Table 1 and available in Supplemental Materials). The majority of the participants focused on their water and soil results. Eight reported that they were on the public water supply and four on private well water. The majority of participants’ actions to reduce exposure after the study were related to drinking water followed by incidental soil ingestion, inhalation of indoor dust, inhalation of outdoor dust, and lastly modifications to their child’s diet (Table 2). The focus on water is primarily due to the ongoing compliance issues with the public water supplier [21]. First, in January 2012 and again in January 2013, UA SRP analyses of drinking water in local homes demonstrated that arsenic was above the US EPA drinking water standard (10 μg/L) [41]. The community used this information to alert the public water supplier and ADEQ to the problem. ADEQ issued a notice of violation to the water utility for exceeding the MCL for arsenic, as well as other regulated contaminants [23]. The public water supplier reported four instances of arsenic exceedances to the community in the Dewey-Humboldt Town Newsletter between 2012–2013. The public water supplier’s exceedance of the arsenic MCL, combined with UA SRP research results, are the most likely reasons why the focus was on water [21]. The participants who were on public water expressed resentment towards the public water supplier:

The Humboldt Unified Water Company that I'm currently spending $75 a month for my water, because I don’t think that I should have to pay for something that isn’t up to par. And if they’re posting signs not to use the water and cooking in and drinking, then I think that maybe there should be some kind of rebate.

We know it’s in the tap water even if you don’t have your own well, and there’s notices all over Humboldt about the—it doesn’t say “Don’t drink the water. Don’t have the water.” It just says “The Humboldt water has a higher level of arsenic this week” or whatever, and they put it in the paper. But they don’t tell you what to do.

One participant mentioned the power of seeing the results, and how receiving his personal water results made a difference:

Yes, I had heard that the water was bad here prior to the warnings that we had gotten from the sheriff's department. But once you see it compared from home samples on your own land that you're living in soil and water tables it strikes you deeper.

With regards to soils and incidental ingestion, participants reported interventions such as removing all family members’ shoes before entering the home, not allowing their children to play in certain areas outside the home, and washing their hands after being outside. One person stated, “And then I see how much is in the soil, so it’s like, Okay now I wanna find out what to do to lower that number”.

Participants reported interventions such as laying rock down and planting trees to reduce outdoor and indoor dust, keeping windows closed during wind events and throughout certain seasons. One participant described a series of interventions:

When you’re sweeping and dusting, try to do a wet dust when you’re wiping things down. Switch out your vacuum cleaner bag more often…. My kids now take off their shoes as they enter the house. So that there’s less dust accumulating in the home.

Additional efforts to reduce exposure are highlighted in Table 4. Interestingly, even though participants did take action to reduce their family’s exposure, eight of the 11 who reported taking action also made statements referring to the notion that there is “nothing I (or we) can do.” Parents mentioned the mine and that the contamination has already occurred (*n* = 5), affecting water and soil quality and that they had to live with it. Subsequent to the water company receiving a notice of violation from ADEQ, users do not think the water company will operate in compliance with regulatory standards. Further, even if the water company eventually did comply with regulations, participants reported that they do not trust the water provider and will not drink it again. In early 2016, ADEQ reported that the local water provider is currently in compliance and are working on disseminating this information. Additionally, a few doubted the ability to truly contain the mine tailing waste:

I’ve got a really good view of that whole thing. So when wind comes through you can see the dust flying. And that dirt back there on the Iron King [mine tailings pile] one is orange. And the one here closest to me [smelter slag pile] is whiter. It looks like ash kind of. So. Even though like 95 percent of the storms and the winds that were always coming from the south and going north…I know that they [US EPA] got a big plan together and they put some kind of stuff over the dust to keep it from blowing in the wind and everything. But that only lasts so long and it starts up again…Is there anybody else besides the EPA? I mean do they bring other people? Do they have people that monitor for them while they′re not here?

### 3.3. Seeking Out, Comprehending, and Evaluating Environmental Health Information

#### 3.3.1. Understandings about Exposure or Health Outcomes

Overall, parents’ understandings of exposure routes were accurate and this is reflected in their intervention strategies (Table 4). Parents even described how MESH opened their eyes to new exposure routes or gave them an opportunity to be part of the science:

Before the MESH study I didn't even think at all that there were metals in the soil and in our water. It just brought to light that there are certain chemicals and metals and things that are combined with our water and our soil and that they can have a—if the levels are raised enough, they can have a negative effect.

Additional exposure routes were mentioned by the parents, such as dermal absorption. Parents ensured that their family drank bottled water or water that had been treated, but they continued to, for example, bathe, wash dishes and brush their teeth with the untreated water. Two parents questioned whether dermal absorption was an exposure route and requested additional information. Participants raised concerns regarding the removal of valuable nutrients due to water treatment processes (i.e., reverse osmosis) and that bottled water did not contain fluoride (*n* = 3). Further, one participant stated the local Woman, Infants and Children office is concerned that children will develop nutritional deficiencies from being on bottled water and that the federal Woman, Infants and Children office views bottled water as an additional risk to children’s health. Drinking water can contribute to a child’s nutritional mineral intake, such as manganese, molybdenum calcium, zinc, copper, zinc, iron, and magnesium [43]. In general, mineral content, such as calcium, magnesium, sodium is greater in tap water than bottled water, but the intake of these minerals is best fulfilled via the consumption of foods in which these minerals are abundant and bioavailable [44]. It is recommended to check the mineral content of drinking water, whether tap or bottled, and choose water most appropriate for an individual/family’s needs [44]. This highlights an opportunity to educate families about where nutritional minerals come from and methods by which to prevent deficiencies.

A unique situation occurred in a case when a child’s urinary arsenic level approached the 95th percentile of the National Health and Nutrition Examination Survey (NHANES, described in more detail below), and the child was re-sampled. MESH researchers questioned whether the high exposure might have resulted from a dietary exposure to organic arsenic via fish. So the parent decided to use this re-sampling as an opportunity to conduct a natural experiment and in the participant’s words “And this was cutting out all the rice—no Rice Krispies, no white rice, no apple juice, no fish, no nothing during this time that would have any kind of arsenic in it and he still came out high.” The parent wanted to be sure to remove all dietary sources of arsenic to determine what may potentially be influencing her child’s urinary arsenic level. Rice is considered a natural arsenic accumulator [45]. In April 2016, the US Food and Drug Administration proposed an inorganic arsenic limit of 100 parts per billion for infant rice cereal [46]. The child’s urinary arsenic level remained above the 50th percentile, but the parent reported a noticeable difference in the toenail sample. Most children in the MESH study had urinary arsenic concentrations above the NHANES 50th percentile and though at this point it is not clear what role dietary arsenic plays in this community as a whole, this example illustrates how a parent further educated herself and actively participated in the resampling design. This parent strongly recommended that the arsenic data be reported back in terms of organic and inorganic, stating “it would have been a heck of a lot easier for me, knowing that, ‘Okay. Well, organic arsenic breaks down in the body a heck of a lot different.’ To put this parent’s comments into perspective, it is helpful to know that ingested inorganic arsenic is metabolized to a dimethylated (DMA) or monomethylated (MMA) form prior to urinary excretion [47] and DMA, MMA, and arsenobetaine (from organic arsenic sources like seafood) are the main contributors to the total urinary arsenic levels [48]. Inorganic sources of arsenic from seafood will differ by community and country, and tend to be lower in Arizona than the rest of the U.S. and much lower than high-consumption counties like Japan [49,50]. Recent seafood ingestion will greatly increase the level of total urinary arsenic and comprise the highest percentage of the total urinary arsenic [48]. Rice consumption has also been related to urinary arsenic concentrations in adults [51] and it has been estimated that children <3 years of age have the greatest exposures to inorganic arsenic primarily due to dietary sources such as rice [52]. Lastly, the presence of arsenic in urine is an indication of recent exposure (within 24 h with a half-life of four days of exposure), while arsenic in hair or fingernails is an indication of exposure within 3–6 months [53]. Parents expressed concern for the future of their children’s health, recognizing that the chronic low-level exposure could lead to health effects in their child’s future. A parent brought the results to their child’s pediatrician and the pediatrician asked to make a copy of the results to keep in the child’s record. Another parent stated they would keep the data on hand in case anything comes up. One parent stated, “If the arsenic or the other stuff is going to affect you, it's going to take at least 30 years. And I’m like, okay, for me, that's one thing, but for the kids, that's their prime time in life. So that’s a big deal”.

In some cases, a parent’s personal and collective environmental history may influence the interpretation of the exposure data [27]. For example, two parents who have lived in the town their entire lives did not express the same level of concern to reduce exposures as the other parents and have not implemented any changes to reduce exposure. As one noted:

When I was a kid, like my mom said, it was there before I was born. We used to go out there. My dad would be doing stuff out there, and we’d be playing around, and—you know I’ve just been around it my whole entire life. And it’s never seemed to bother anything.

It is likely that their life-long residence has made them more willing to accept the level of contamination, perhaps because it normalizes the hazard.

#### 3.3.2. Report Back Styles and Comparisons Made by Parents

Nine participants were asked what report they preferred, the individual (just their family) or the summary (aggregate of all results). Six participants preferred the individual booklet and two preferred the summary booklet. When participants were further asked if they wanted both the individual and summary packet, six stated yes. One participant highlighted that the researchers could provide the individual results sooner, so parents could implement methods to reduce exposure as soon as possible. When asked what they preferred, responses varied, for example “I’m not trying to be selfish, it’s just that I wanted to know specifically about my family.” Four participants advocated for both report back types, recognizing that the individual booklet was personal and the summary allowed them to see “…where we fit in the study with everybody else”. Data on the remaining six participants is not included because they did not have the summary report for comparison. The following quote illustrates the reason for individual report back preference:

I liked the individual reports. It was easier for me to see the information that I wanted. In the final summary I can compare to other people but, ultimately, I don’t really care in a personal sense. It's interesting in a scientific sense for me to know what’s going on in the area. In this area I wanted to know what the exposure was in our daily routine.

#### 3.3.3. Comparing Siblings

When a household had more than one child in the study (*n* = 6), parents compared the children’s levels and explored why one child’s levels differed from the others. Using the individual household booklet, they would begin to compare and contrast the activities of their children to determine what could be contributing to that differing concentration, for example:

She’s my oldest…she’s not outside as much as the younger ones. But is it concerning to me that her levels are higher than theirs in regards to arsenic. It’s almost double or triple the amount that the two younger ones are at…that’s probably the main concern really.

The lead in my daughter… Hers was raised, compared to my younger ones. And she's not really outside that much compared to the activity that they are outside. But that's probably the main concern really, the main question that I had was because why is hers, her level higher than theirs and she's not out as much as they are.

This says that my son, the oldest child that went to the study, has higher levels of arsenic than the other two. I have no idea why that’s the case. They’re all exposed to the same air, water, and dirt. Possibly, now that I’m analyzing it, it has something to do with the fact that he takes insulin. He’s diabetic, or maybe it’s the other way around, or maybe that’s why he’s diabetic. We don’t know what causes diabetes.

(One parent talking to another): Remember when we talked about that, because she went to school, he stayed home. That’s going to have somewhat of a big difference because she was away from the house, she wasn’t, I don’t know—it’s kind of a big difference, because she was at school.

#### 3.3.4. Comparing Across the Study

When comparing their children’s results to the rest of participants, parents would worry when they were “greater than the rest of participants” and not be concerned about the data when they were below the majority of those in the study. This reaction occurred for both the biomonitoring and environmental samples. Additionally, this reaction was similar to how parents compared multiple children in their household. In a few cases, parents would see if the child in their home was still on the “high end or higher than” the other participating children. This comparative behavior has been previously documented when a participant discovers that the level in her home falls below the study average, she may interpret her result as “safe,” whereas another participant may be concerned to find his value falls at the upper end of the study distribution, even if the entire distribution falls below a benchmark [6].

#### 3.3.5. Environmental Samples

For the environmental samples, parents were quick to determine whether they were above the MCL for water or the AZ soil remediation level for their outdoor soils and indoor dust and relied heavily on these values to understand their household data. Parents viewed the water as the most important exposure route and tended to focus on whether their water was above or below MCL. In general, parents interpreted the environmental data more easily than the biomonitoring data. This may be due in part to existing environmental quality guidelines/MCLs and familiarity. There have been past community-engaged research efforts in the area where participants and non-participants received multiple informal science learning opportunities and environmental monitoring reports related to their soil, water and vegetable quality [25] and ongoing announcement by the US EPA have been made about environmental sampling efforts.

#### 3.3.6. Desire for Further/Future Studies

When parents were asked, “Are you glad to have learned about the sampling results for your own home?” and “Would you participate in another study like this again?” all parents stated yes. This interest may, in part, be due to participant bias. For example, those who were interested in being interviewed may be more eager than others to participate in more studies. One stated, “I just like seeing the results…like getting a report card.” Parents who had done some type of intervention were eager to know whether their intervention worked to reduce their children’s urinary arsenic levels. Additionally, some wanted future water monitoring to ensure the public water supplier was really fixing their system and providing water that met regulatory standards. Other questions for future studies were related to: whether their child’s urinary arsenic would increase as they continue to live in the area, determining the relationship between the mine and the concentrations observed in the MESH environmental and biomonitoring samples, and whether some areas are worse than others, and if so, is this related to the mine or naturally occurring? A few parents (*n* = 2) wanted a general health study of the entire town to understand what types of diseases are prevalent in the area. Additional information was requested such as: ‘What are the specific health effects associated with arsenic and heavy metals exposure?’, ‘What can I do about my child’s exposure now?’, ‘How can I remove the arsenic from my children now?’ (*n* = 3). One parent stated:

I want to look into it and see if there’s any long lasting results for my children is mainly the thing I want to—you know is there anything I can do? Other than obviously by knowing this I’m gonna change how they play outside and, you know the water they’re drinking and all that kind of thing. But is there anything else I do to kind of help with what they’ve already been exposed to?

### 3.4. Challenges Reported by Participants

#### 3.4.1. Additional Inquiries

Although participants had a report back preference as described above, five requested a face-to-face sit down to with a researcher to go over their data, stating “I think I would have had more [of an] understanding if someone was there personally that I could talk to more one-on-one.” It is important to note that the MESH project manager called every participant to be sure they received their packet and to discuss the results. Perhaps this subset of interviewees desired more one-on-one experiences and this might be why they agreed to an interview that focused on their personalized experience. Only one participant did not discuss their results by phone with the MESH project manager. Participants relied heavily upon this phone conversation with the MESH project manager. They trusted the MESH project manger’s interpretation and felt comfort in whether they said their child was okay or not. It was clear that having a conversation with the project manager was a major source of clarity and they relied on this phone conversation to make sense of their data. Most participants reported that they would call the project manager if they were not sure what the results meant. Participants reported conversations with the MESH manager as pleasant and informative, for example:

That was why I asked [MESH manager] when he called. He said “what don’t you understand” and [participant responded] “is there anything in there that we need to worry about?”.

I was going back and forth. But still, I know that I didn’t have all of it 100 percent in my head. So, you know when I went over it on the phone with [MESH manager] and at that time I remember him explaining it and I was understanding it better.

When participants were asked “What was or was not useful about the way the study results were reported?”, responses were related to wanting more details related to each metal reported, risk data and interpretation, and a spatial representation of the data. With the exception of arsenic, the MESH reports (individual and summary) did not give specific explanations for each metal they measured. Participants wanted to know: What is each metal and why measure it? (*n* = 5); What is the risk associated with their child’s urinary arsenic level and should they be concerned? (*n* = 6); What is the health effect of having the metal in your body? (*n* = 5); and How do I get this metal out of my child? (*n* = 3). These additional questions indicate a desire to learn more and request for more data. The following responses reveal these additional data requests:

All I can think of is how much is the maximum you can have without it hurting you.

What is bad? What is the risk at that level? What do I do if it’s something that’s above the standard? What do I do?

I would say in some of the metals, maybe explain what the metal is. And maybe if it has a negative effect on your body.

I understand what lead is, but there were other things that were different names that I’d never even heard of before, and I didn’t understand what they were…The elements should have been described, explain how the children could be in contact with the element and what each element is.

Now that you know your results, here’s what you can do for your kids. That would be kind of neat.

Before I talked to [MESH manager] I opened it up, I looked at it, and I go, “What the heck? What is this about?” I read this over here, yes, and then, of course, I put two and two together, but there are thousands of questions on my mind. How’s it going to affect a four or five-year-old child? What’s the effect of his brain growing, his body growing, and what’s he going to do? How can we flush his body, get all those metals out? Something like that. Go in depth, especially when children are involved.

One set of parents noticed their beryllium levels were elevated in water and stated “the concern is what is beryllium? And then what’s the effect of beryllium? And then what can we do to ourselves to protect us from it?”

#### 3.4.2. Physician Knowledge and Advice

Five parents reported that they shared the results with their child’s pediatrician and the next steps taken by the physician varied. In one case the doctor only focused on the blood lead levels and questioned why the study was conducted in the first place. Another doctor thought it was interesting, and while he or she did not facilitate a discussion about the MESH results, still decided to put it in the child’s record. In one case the child’s urinary cadmium levels were above the NHANES 95th percentile and the parent reported that the pediatrician was not worried at all. Although the child’s urinary cadmium levels were well below occupational safety standards, the health implications for a child are not well understood. Cadmium is a known reproductive toxicant thought to disrupt hormone production throughout sensitive developmental windows [54], and it could also be an indicator of iron deficiency [55]. During chronic exposure, cadmium accumulates in the liver and kidneys and the estimated half-life of cadmium in the kidney is from one to four decades [55]. The parent was still concerned and wanted to seek help from a homeopathic doctor to identify remedies to get “rid” of the heavy metals in her child’s system. The remaining participants stated that the doctors thought the data was interesting, but were not concerned or worried in any way. In general, it appeared that the pediatricians were not equipped to deal with the MESH dataset. Before the study began, the MESH research team completed a mass mailing to pediatricians, family practices and general physicians within the town of Dewey-Humboldt as well as the neighboring towns of Mayor, Prescott Valley and Prescott to notify physicians in the area of the study and received no response. Later, the MESH project manager reported that after the study began they learned that families would travel 1.5 h (84 miles) to Phoenix, AZ, the closest city, to see a physician. These observations echo the challenges associated with rural populations, as well as the barriers that may exist preventing physicians from adhering to preventive service guidelines. Rural physicians reported that barriers to preventive practices are due to their own focus on occupational risk factors and that patients are discouraged by not having adequate access to resources and specialists and by having to travel greater distances to health services, which reduces their frequency in visits [56]. Additionally, these observations suggest that physicians, especially pediatricians, need to know more about positive links between environment and health and need additional environmental health training [57,58].

#### 3.4.3. Comparative Values

NHANES, which reports on chemical exposures in a representative sample of the US population, was used in the MESH study for comparison. The MESH researchers used the 50th and 95th percentile of the metals from NHANES participants aged 6–11 years in 2009–2010 to frame the children’s results. Though the MESH report-back material explained the definition of the NHANES values, MESH participants on average used the NHANES values as standards or regulatory benchmarks. Families reported being under the 50th percentile as being ok, between the 50th and 95th percentile as high or not ideal, and then above the 95th percentile as unacceptable. While comparison to values like NHANES are useful to provide some reference, these comparisons have the potential to lead participants to over- or under-estimate risks or to misinterpret reference group levels as safety benchmarks [59]. To clarify the interpretation for participants, research teams should define the reference levels used to ensure that they are not confused with regulatory benchmarks [7] by spending more time with participants describing NHANES and the definition and application of percentiles. In past biomonitoring studies, researchers used the NHANES values when there was not an established reference value and/or scientific uncertainty surrounding toxics. In the case with arsenic there is a biological exposure index (BEI) of 35 µg/L (inorganic arsenic plus methylated metabolites in urine) [60], but the MESH research team decided that this BEI was not appropriate for children.

In combination with the NHANES values, participants evaluated whether they were “good or bad” based on their conversation with the MESH project manager or toxicologist (described above). MESH researchers reported in the summary of results that “the arsenic level in urine was above the NHANES 50th percentile for 56 of 68 children (82%) in MESH, compared to a group of 68 typical children, in which only 34 of 68 children (50%) would have arsenic levels in urine above the NHANES 50th percentile.” For example, one parent remarked:

In terms of the standards of what it should be, our kids are well within that or well below that. One of them was about the 50th percentile and one of them was below it. That’s the 50th percentile so they’re average, what the average person is. I still was the same. Why is there arsenic in their urine? I don't want any arsenic in their urine but then I thought about this is still pretty standard or average for most people. That’s the way I thought about it.

Two participants suggested graphically representing the data in a bell-shaped curve or other graphic to show that “here is the national average, here is where your child is at, here is where the other children are…” Where there were no NHANES values (i.e., aluminum, beryllium, chromium, and nickel) or regulatory benchmarks (i.e., there are no Maximum Contaminant Levels in drinking water for aluminum or nickel), several participants compared themselves to other children in the study to make sense of the data. Very few participants mentioned or discussed the toenails data and we anticipate this may be due to the lack of reference values.

#### 3.4.4. Reporting Data Spatially

The desire for a map or some sort of spatial representation of the data was mentioned (*N* = 6) and when the interviewer explained the IRB and protection of privacy, four out of the six stated they were willing to forgo their privacy for a map to demonstrate the distribution of homes that participated in the study, “I wouldn’t mind because it’s for a benefit of everybody in this area so it wouldn’t have bothered me.” The remaining two participants did not further elaborate, besides wanting the map. They wanted to further understand how their values compared to others and where they were physically in comparison to other households in the study. Sacrificing privacy for the data visualization was noteworthy and participants had questions that they felt a map could answer. They did not care if others knew who they were, or where they lived—they wanted to get a better sense of the impact of the site, the extent of contamination/high endemic areas. “If you are going to compare the values, then tell me where they are” and “Why is my concentration for arsenic in house dust greater than others?” One participant stated, “It may encourage people to get tested also if they see some of these people around us—if they could put it out in the newsletter or whatever it may be. It may encourage more participants.” Indeed, resident and citizen science groups that have organized around environmental contamination often engage in “lay mapping” which they make public, in order to demonstrate the geographic clustering of cases [61]. Though these six participants are advocating for a map, there may be other participants (specifically those who did not participant in the PERE study reported here) who might not want their data shown or have their identity revealed. Also, there may be future unintentional consequences to generating a map (e.g., financial, decrease in property value) beyond the study and this could discourage future biomonitoring studies, especially those coupled with an extensive results communication process.

#### 3.4.5. Resource Networking

A component of EHL is to develop a wide range of skills and competencies in order to seek out, comprehend, evaluate, and use environmental health information to make informed choices and to move beyond individual behavior and focus on a range of social and environmental interventions at the community level. When participants were asked whether they have considered any community action to reduce exposures, seven responded, although only two reported actually doing something. One started a community organization, the Community Coalition of Dewey-Humboldt, after the Iron King site was added to the US EPA’s National Priorities List (Superfund) in 2010, to be able to then apply for an EPA Technical Assistance Grant. Those grants provide up to $20,000 for affected residents to hire their own technical consultants to help understand and interpret the highly technical material presented in EPA clean-up protocols, and are therefore a central component for advancing EHL. That participant was also hired as a Field Technician for the MESH study, demonstrating an important benefit to community members of being involved in a community-engaged research project. The other reached out to parents at a school. Four stated that they were willing to do something if: (1) their household data was “more shocking”; (2) it was directed towards the public water system, stating what is there to do about the mine, that is the past; and (3) if they cared for the town council members. One stated that they felt “…the community as a whole is too deprived…I don’t think they would support it even if we—a group got together and tried to start something like that.” Unfortunately, MESH participants rarely interacted or engaged with one another. MESH did host a total of three meetings (one open only to participants, two open to general public), but they were poorly attended; only three participants reported attending a meeting. This lack of interaction among participants was a missed opportunity for both the community and researchers. In past result communication studies,, community gatherings were reported as being rich in interaction [28] and a place for participants and nonparticipants to engage in discussion, meet one another, and identify others who were experiencing similar exposures [26]. Timing and competing family priorities might explain less community interaction and participation in community gathers during the MESH study. It might have been hard for families to find the time to attend a community meeting. To increase participation, MESH researchers had university students attend the meetings to provide childcare. As stated in the methods, a CAB was initiated to gather participant feedback and this may have served as a supplemental mechanism for participant interaction. In the study described here, three mentioned wanting to know how other families were dealing with the data, whether they were doing anything to reduce their exposure and if they were, was it working. One participant who did her own research and laid rock to reduce dust stated, “I would be curious to know what everybody's doing because we can all learn from that.” Another participant, having a background in construction, knew to put trees around the property and cover barren soil with rock to reduce dust and could have shared such techniques.

## 4. Discussion

By evaluating EHL within an EJ community neighboring a hazardous waste site, we have demonstrated that reporting back exposure and biomonitoring data can go beyond simply an act to ensure knowledge acquisition to an action-based effort that increases EHL. The extensive report back process reported here provided individuals and communities with the tools and information to exert control over the environmental exposures that may lead to adverse health outcomes. The report back process reduced information disparities and promoted EJ. Increasing EHL can improve and support community-based environmental health initiatives, such as, but not limited to the US EPA’s for addressing community environmental health concerns [62] and the CDC’s Protocol for Assessing Community Excellence in Environmental Health [63], both are proposed practices designed to advance environmental health community-government partnerships.

In addition, we hypothesized that such documentation of potential learning and action using a free-choice learning framework would be an effective method by which to assess the report back efforts in an EJ community and, in effect, evaluate a community member’s EHL related to metals in the environment. In terms of advancing the field of EHL, we aimed to assess the following put forth by Finn and O’Fallon (2015): (1) the relation between EHL and resilience; (2) whether messaging about environmental factors actually lead to behavioral change; (3) whether messaging leads to prevention, reduction, or mitigation of environmental risk factors; and (4) different approaches for measuring success. Key findings were that participants used the data to cope with their challenging circumstances, the majority of participants described changing their family’s household behaviors, and participants reported specific interventions to reduce families’ exposures. The strength of this study is that it has given us insight into what people learn and gain from such report back efforts, what participants want to know, and what type of additional information participants want. This study has also demonstrated that different modes of results communication such as multiple reports with written materials and data visualizations, community meetings, face-to-face conversations, and phone calls are critical to environmental exposure results communication. Further, consulting with the community on report back layouts can leads to improvements in communication efforts. The CAB and collaborating with study participants allowed for a better design of report back materials and take home messaging. For example, in the summary of results packet, CAB members advocated for the dot figure (Figure 2) and the simplified infographics (Supplemental Materials). This information can help inform and improve future report back efforts conducted by other environmental health researchers.

### 4.1. Study Limitations

A limitation of the study is that the MESH participants interviewed were a subset of those who agreed to be recontacted during the MESH informed consent process in 2012. A total of 26 out of the 34 participants stated that they could be contacted for future studies (recontacted). Of the 26 participants, 17 said a PERE researcher could contact them to schedule an interview, one declined, and for the remaining eight, the MESH manager was unable to reach them. Of the 17 who agreed, 14 interviews with the PERE researcher were completed. Thus, we cannot be sure that the exposure experience we characterize here represents participants who declined to be re-contacted in the original MESH consent. A minor limitation is that there was a time lapse between telephone notifications (from MESH toxicologist or manager) and individual results packets (May 2013–January 2014) and the summary of results (summer 2014). The MESH project manager timed the summary of results packet to arrive approximately a week before the PERE interviews were conducted in summer 2014. This time lapse between the individual and aggregated data may have affected participants’ reporting of their initial experience. Though there were time lapses and the interviews were conducted around a week after the summary of results packet was received, we are confident that are methodologies captured lasting behavioral changes. Since the inception of MESH in 2012, exposure reduction information was provided and this messaging was consistent throughout the project. For example, MESH researchers presented to Gardenroots: The Dewey-Humboldt Arizona Garden Project citizen scientists in June 2011 [26]. Lastly, this study assessed self-reporting of action versus a biomarker measurement and this may be perceived as a limitation. It might be beneficial for future communication and intervention studies to perform a post biomonitoring sampling to observe any reduction in contaminants of concern (e.g., [64]).

### 4.2. Implications for Future Report Back Efforts

#### 4.2.1. Improving Health Care Practitioner’s EHL and Involvement in EJ Communities

When parent’s decided to discuss their child(ren)’s MESH data with their pediatrician, responses were inconsistent and in some cases, in adequate. Physicians, in this case pediatrician’s need to receive more training in environmental health. A good resource for trainings and continuing education opportunities are available through a network entitled: “Pediatrics Environmental Health Specialty Units”. This network is a resource for public health professionals, clinicians, policy makers, and the public to get more information about the impacts of environmental factors on the health of children and reproductive-age adults [65]. Arizona is located in region 9 and currently there is a one regional contact listed who resides in California. Based on the outcomes of this study, combined with the ubiquitous mining (active and legacy), natural geology, and number of rural communities in Arizona, we need an Arizona-based Pediatrics Environmental Health specialist.

#### 4.2.2. Mapping Data and Privacy

The fact that people were willing to sacrifice their privacy for the sake of data sharing and to advance their understanding of the results is to the best of our knowledge the first time this perspective has surfaced in our evaluation of biomonitoring studies. This request challenges existing IRB approaches to confidentiality, which prohibits participants from knowing who other participants are or by providing spatial data that would easily identify participants to each other and to non-participants. With regards to autonomy, during the informed consent process, in addition to asking participants if they want to receive their results, perhaps participants can also be asked whether they want to see their data, along with other participants on a map or other geographic representation of the results and to what extent they want this data shared. Meaning, participants could specify to only have the map in the summary of results packet and available only to other participants and/or have the map with the aggregated data publically available. Participants may be asked to what resolution or what format the map should be drawn, for example one participant recommended using a Google map with general pinpoints, another suggested creating a map with the Iron King mine in the center and then drawing radial lines at different distances from the mine itself. With regards to beneficence, the risk of being identified in a study may be considered a risk and clearly describing the risks and benefits of a map would need to be described. One participant stated:

You can know where I live and what my exposure…it is not necessarily super private information. It′s not like I′m telling people my personal medical information…Anyway, I personally would consent to being a dot on a map and the details of that even if it did disclose that I participated in that study or that I was part of it.

With this in mind legal issues may need to be addressed. For example, a map showing the concentration of contaminants in or around their home may impact the sale of a home, although there are already multiple ways in which to determine a home’s proximity to contaminants or hazardous waste. For example, the general public may access the:

- US EPA’s “Superfund Sites Where You Live” web page and see where the US EPA is managing the clean-up of uncontrolled or abandoned hazardous waste sites [66]
- US EPA’s toxic release inventory program, which tracks the management and release of certain toxic chemicals [67]

Furthermore, states may use mapping to manage hazardous waste sites and programs, brownfield redevelopment initiatives and implement water monitor programs. Using Arizona as an example, ADEQ conducted a study and mapped the concentrations of arsenic and other chemical of concern in groundwater [36]. Though the resolution of the map is low, one can clearly infer from the data which areas may have elevated levels of naturally occurring arsenic in their private well or that the small water provider in that area may struggle to provide water that meets the regulatory standard for arsenic. These above examples demonstrate that there are already maps that exist depicting environmental monitoring data and IRBs may need to reconsider community map-making efforts as a means to advance EHL.

#### 4.2.3. Reporting Data Back in the Context of Risk and Health Outcomes

Throughout the MESH project, the research team specified, “MESH is an exposure study, not an epidemiology study. An epidemiology study attempts to explain the relationship between an exposure and a health outcome in a defined group of people… MESH is an exposure study designed to find out if people are being exposed to metals through soil, water, or dust and how much exposure they may be getting.” The MESH team was particularly interested in how the biomonitoring levels compared to the rest of the nation and to determine if a health study was necessary in this community. MESH researchers repeatedly stated that the study was not designed to define specific health outcomes. The study was designed to determine if the communities’ exposures were high enough to warrant future health-related studies. Nonetheless, participants requested additional information regarding risk and potential health effects associated with the exposure data and this is a typical request from communities neighboring contamination and in other types of biomedical studies. This request and need has been identified as a challenge associated with interpreting and using biomonitoring data [6]. Here are some suggestions on how to address inquiries about risk and possible health outcomes in report back efforts:

- Cancer Slope Factor-Based upon previous epidemiological studies, the US EPA has established a reference dose (RfD) and cancer slope factor (CSF) for arsenic. The CSF (1.5 milligram/kilogram, body weight-day) may be used by regulatory agencies to estimate an increased cancer risk from a lifetime oral exposure to an agent. Perhaps the CSF could have been used to interpret the data and provide a frame related to risk. For example, the concentrations observed in the environmental samples (water, soil, dust), combined with the child health questionnaire data on exposure (e.g., ingestion of water, duration out/indoors), could have been used to estimate the child’s average daily dose of arsenic, and then this value could then be compared to the CSF. If a CSF is available, it may be useful to use them, but only with an explanation of how these values are developed and with a clear description of the assumptions that go into an exposure assessment. Though a CSF could have been used for the MESH study, in general, few health-based values (such as a RfD, CSF, reference concentration or BEI) are available to put biomonitoring results into context [5].
- A Transparent, Bi-directional Discussion about Risk Assessment with Communities—Current knowledge of exposure and dose is limited for most contaminants of concern and since the human health risk model is sequential, the lack of accurate information can severely weaken the ability to assess risk and protect human health [68]. These data gaps are more pronounced in children’s exposure assessment (e.g., [69]). A more holistic approach is necessary if risk assessment is to remain a relevant and reliable decision-making tool [70]. Efforts are needed to improve cumulative (multiple chemicals) and aggregate (multiple routes) exposure and dose modeling and children’s exposure assessments need to be further informed by physiologic characteristics (e.g., absorbed dose), behavioral development (e.g., manual dexterity), hand-to-mouth frequency (e.g., [71]), physical activities, diet and eating habits, gender, socioeconomic status (SES) and race/ethnicity [72]. A risk assessment that clearly defines the gaps and limitations of the current paradigm, in addition to NHANES data, may provide an additional level of learning, offer another way to interpret exposure data, and further assist a community member to translate the results into action. Reporting data back in terms of risk may prepare and equip communities neighboring state and federal Superfund sites with the “risk language” in order to then critically assess cleanup decisions [25]. It is recommended to initiate a risk assessment dialogue with communities neighboring contamination beyond the one-directional traditional model. This dialogue should not only describe the inherent difficulty of establishing the existence of a causal relationship between measured concentrations of environmental pollutants and health effects, but more importantly, to work with the community to more accurately define the characteristics that influence a child’s exposure. Children themselves (ages 8 and up) can report their activity patterns and diet and eating [73] and this would facilitate informal science learning. Families can work together to inform exposure parameters related, but not limited to behavioral development and the role socioeconomic status and race/ethnicity play in their child’s exposures.
- Provide Other Empirical Data Sets for Reference-Another suggestion would be to frame participants’ results in terms of other empirical data sets and this suggestion echoes a MESH participant’s recommendation to add international data to the report, and specifically referenced Bangladesh (known for high arsenic levels in drinking water). Researchers could provide the data from larger epidemiological studies and the type of health effects that were associated with those findings. For example, a table or infographic depicting population size, sources of pollutants (in this case arsenic), exposure route(s), environmental and biomonitoring data, identified health effects, and other relevant factors related to the population (e.g., SES) from studies completed in highly exposed populations such as Bangladesh (e.g., [74]), Chile (e.g., [75]), Mexico (e.g., [76]), and Taiwan (e.g., [77]) and those that have been exposed at relatively low levels (e.g., [78,79,80]). These studies could be presented to participants to address their interest in health effects and risks associated with arsenic exposure, such as increased risk of cancer, cardiovascular and respiratory conditions, and diabetes mellitus [79]. This approach would frame their exposure by referencing other large-scale studies and it would further participants’ EHL and understanding of exposure science and epidemiology, shed light upon the studies used to generate reference doses and CSF, and further emphasize that more research needs to be focused upon low-level chronic exposure in children. In contrast, there is a chance that this comparison could make people think they are okay since others are worse off. By providing the data from previous epidemiological studies, including both those with high and relatively low exposure levels, we anticipate this will not be the case and that participants will observe the range of exposures and what type of short- and long-term health effects are associated with the contaminant of concern and exposure pathway. Overall, this approach will provide another opportunity to promote EHL.

## 5. Conclusions

Our study illustrates that a biomonitoring study coupled with extensive report back process can increase environmental health literacy in an environmental justice community. Participants comprehended and evaluated their children’s data, used the environmental health information as a foundation to ask new questions, took action to reduce their family’s health risks, and used the exposure data to make informed choices. Much attention was given to the water quality and several MESH participants expressed resentment and frustration with their local water supplier for serving water that exceeds drinking water standards. One provided a policy intervention and suggested holding the water provider responsible; requesting they should issue rebates/refunds to families who paid for contaminated water. This notion is being demanded in Flint, Michigan and across the entire country.

When asked about the utility of the report back process, participants suggested novel ideas regarding how to improve future report back efforts and increase community networking. Though it was clear that MESH was an exposure study, participants did request risk assessment and health impacts data. Efforts should start considering how to frame exposure data in terms of risk and health outcomes and practitioners should initiate a transparent, bi-directional discussion with study participants to improve the overall environmental communication process.

## Figures and Tables

**Table 1:** Contents of result packets provided to participants in the MESH study.

1st Results Packet-Individual Report	2nd Results Packet-Summary of Results
Cover letter—One-page narrative describing the goals of the study and key information about the MESH design List of important terms (glossary) A set of seven graphs per page. Pages were dedicated to: Environmental media, “Metals in your water, soils, and dust” showing the participant’s sample in comparison to existing standards (ADEQ soil remediation levels, regulatory maximum contaminant levels in drinking water) Biological samples, “Metals in urine, toenail” showing the participant’s sample in comparison to NHANES 50th and 95th Percentile (creatinine corrected concentration) References values for the chemicals and where to get more information about the references used in the report Additional information page with a list of websites (e.g., Agencies for Toxic Substances and Disease Registry Toxicological Profiles, ADEQ, US EPA) to learn more	Cover letter—One-page narrative describing the goals and design of the study, and package content One-page narrative summary of background, key results and where to get more information 8-pages summary of MESH findings (including results interpretation and what it means to participants) 3-pages summary containing pertinent information about MESH and environmental exposure studies in general (e.g., what they are/are not) List of important terms (glossary) Interpreting your results section and example chart on how to read the graphs Graphs of participant’s data compared with other homes in the study, along with the following reference values: Biological samples—NHANES 50th and 95th Percentile for urine and blood data (when available) Environmental samples—Regulatory MCLs for water and AZ ADEQ remediation levels for soils References values and where to get more information about the references used in the report Additional information page with a list of websites (e.g., Agencies for Toxic Substances and Disease Registry Toxicological Profiles, ADEQ, US EPA) to learn more Informational brochures: “How to Reduce Your Exposure to Arsenic and Lead in Dewey-Humboldt, Arizona” and “Arsenic in Drinking Water: What You Need to Know” developed in collaboration with the ATSDR Region 9 and AZ Department of Health Services, and the UA SRP

**Table 2:** Actions participants engaged in after the study to reduce their family’s exposure.

Exposure Pathway	Intervention
Ingestion, Potable Water *n* = 11	Using bottled water (10) Working to install a water treatment system to remove arsenic and heavy metals (1) Maintaining current water treatment system (2) Buying products that don’t have chemicals in them to protect groundwater contamination via septic tanks (1)
Ingestion, Incidental Soil *n* = 8	Limiting/reducing/not allowing children’s play time in yard soil (2) Wearing shoes when outside (1) Washing their hands (4) Postponing gardening to build raised beds for garden and play area with imported soils (1) Setting up play sets on something so they are not directly playing in soils (1) Removing shoes as they enter the house (5)
Inhalation, outdoor dust *n* = 3	Covering barren soils with rocks, gravel (3) Planting trees (1) Planting ground cover-grass, clover (1)
Inhalation, indoor dust *n* = 5	Removing carpet and putting in the wood floors (1) Keeping windows closed (2) Vacuuming and/or sweeping more frequently (3) Dusting, wet dusting (3) Switching out your vacuum cleaner bag more often (1)
Ingestion, Food *n* = 3	Eating organic (1) Reducing sources of arsenic from diet (rice products, apple juice, fish) (2) Growing certain vegetables in home garden that do not accumulate arsenic (1)

**Table 3:** Demographic data.

Variable	Not Included in PERE		Included in PERE		*p*
	*n*	%	*n*	%	
Adult Annual Income					0.99
$0–19,000	14	48	12	44	
$20–39,000	5	17	6	22	
$40–59,000	3	10	3	11	
$60–79,000	4	14	3	11	
>$80,000	3	10.3	3	11	
Educational Attainment					0.51
Less than High School	4	11	3	11	
High School/GED	10	29	6	21	
Some College or Vocational School	12	34	11	39	
College	3	9	3	21	
Graduate Degree	6	17	2	7	
Race/Ethnicity					0.88
Asian	1	3	0	0	
Hispanic	2	6	1	4	
White	32	91	26	93	
Households Below Poverty Level	8	50	5	36	0.48

**Table 4:** Comparing children’s urinary inorganic-related arsenic concentrations of those who were included in PERE and those who were not included.

Variable	Arsenic Micrograms Per Liter (μg/L) ^1^	Not Included in PERE		Included in PERE		*p*
		*n*	%	n	%	
>MESH 50th percentile	10.4	20	52.6	16	50	1.00
>MESH 95th percentile	29.4	4	10.5	2	6.3	0.68
>NHANES 50th percentile	5.36	32	84.2	27	84.4	1.00
>NHANES 95th percentile	13.4	14	36.8	10	31.3	0.80

^1^ Inorganic-related arsenic species included (arsenic III, arsenic V, dimethylarsinic acid, and monomethylarsonic acid). These concentrations are the 50th and 95th percentiles for MESH [34] and NHANES for age group 6–11, survey years 2011–2012 [40], respectively.

## Supplementary Materials

The following are available online at www.mdpi.com/1660-4601/13/7/690/s1.

## Abbreviations

The following abbreviations are used in this manuscript:

ADEQ	Arizona Department of Environmental Quality
CAB	Community Advisory Board
CDC	Center for Disease Control
MESH	Metal Exposure Study in Homes
NHANES	National Health and Nutrition Examination Survey
PERE	Personal Exposure Report-Back Ethics
US EPA	United States Environmental Protection Agency
UASRP	University of Arizona Superfund Research Program
