# Supplementary material for: Improving Environmental Health Literacy and Justice through Environmental Exposure Results Communication
Source: Int J Environ Res Public Health. 2016 Jul 8;13(7):690. doi: 10.3390/ijerph13070690 (PMC4962231; doi:10.3390/ijerph13070690)
Supplement: Supplementary file 1 [file ijerph-13-00690-s001.pdf]

MESH

Metals Exposure Study in Homes

Your Environmental & Biological Sampling

Results

Superfund Research Program

Superfund Research Program
The University of Arizona

The University of Arizona

Friday, June 17, 2016
<<Name>>
<<Address>>

Dear <<Name>>,

Thank you for participating in the University of Arizona (UA) Metals Exposure Study in Homes (MESH). Enclosed are the test results of your home's water, soil, and dust and your children's urine, blood, and nails. All of these samples have been tested for seven metals: arsenic, lead, nickel, aluminum, chromium, cadmium, and beryllium, with the exception of your children's blood, which was analyzed only for lead.

In this packet we've included basic but important information about MESH as well as a glossary of terms that describes information you will find in your results.

We are trying to understand how much you and your family are exposed to metals. We are also trying to understand the various pathways by which metals can enter the home. Results from this study will help develop better ways to protect homes and families from metal contaminants. Please keep in mind that the methods used in this study were designed for research purposes only and do not necessarily follow U.S. EPA or Clinical Laboratory Improvement Amendments standard methods. Once the study is completed, we will provide you with another results packet summarizing the overall findings of the study.

If you have questions, please call (520) 626-7470 or email klimecki@pharmacy.arizona.edu. You can also contact Nathan Lothrop at (877) 535-6171 toll-free or lothrop@email.arizona.edu. Thank you again for participating and making this project a success.

Sincerely,

Walter T. Klimecki. DVM, Ph.D.
Associate Professor of Pharmacology and Toxicology
University of Arizona

Important Information about MESH

MESH is an exposure study, not an epidemiology study.

An epidemiology study attempts to explain the relationship between an exposure and a health outcome in a defined group of people. For example, large-scale epidemiology studies established that smoking cigarettes (an exposure) increases the risk of lung cancer (a health outcome). An exposure study is designed to determine and understand the contact a person has with an environmental factor. MESH is an exposure study designed to find out if people are being exposed to metals through soil, water, or dust and how much exposure they may be getting. Though MESH did collect some health information in a questionnaire, this was done to see if health outcomes should be assessed in a future study.

MESH is designed to help us understand which factors will contribute to increased metals exposures.

This study is investigating housing characteristics, behavior routines, general demographic information, and several other factors to see how these may impact the levels of metals in your home. After the study is complete, we will use this information to provide suggestions to participants and other community members about what they can do to reduce their exposure to metals.

Metals occur naturally in the environment.

Some areas have naturally higher levels of metals than others. Furthermore, while we can measure the level of a metal in certain substances, like water or soil, it is difficult to know the original source of the metal. For example, arsenic may be found in soil in many parts of our state because it naturally occurs in these regions. Similarly, some drinking water wells may have high arsenic concentrations because the rock surrounding the well has arsenic in it. Arsenic can also be found in some foods and drinks and in older, outdoor-treated wood. One objective of MESH is to determine if living closer to the mine and smelter tailings is associated with higher levels of exposure to certain metals for residents in and around Dewey-Humboldt. However, we cannot begin those analyses until all sampling is finished. We do not know yet if, and to what extent, the Superfund site contributes to metals exposure in people.

Single exposure measurements are “snapshots in time”.

We are measuring metal concentrations at a single point in time. Measuring metal concentrations with a single sampling does not establish what the exposures were in the past or what they may be in the future. Some exposure measurements can only reflect exposure over

very short time periods. For example, arsenic testing in urine only reflects arsenic exposure over the past several days. Likewise, metal concentrations in house dust may change from week to week, depending on the source and the frequency of vacuuming. As a result, measurements taken only once may not give an accurate picture of past (or future) exposures. This is important, because many chronic diseases associated with environmental exposures only happen after long periods of exposure.

Where available, we have used NHANES measurements as points of reference for urine metal concentrations.

The National Health and Nutrition Examination Survey (NHANES), is a U.S. Centers for Disease Control and Prevention program that examines about 5,000 people per year to assess environmental exposures, health, and nutrition status across the United States. Where NHANES data is available for metal concentrations in urine, we have provided reference values for the 50th and 95th percentile of the children surveyed. The 50th percentile value means that half the children between ages 6 and 11 surveyed had urine metal measurements below this value and half were above that value. The 95th percentile value means that only 5% of the children had urine metal concentrations higher than that value. These percentile values are not health standards or guidelines; they simply show the distribution of urine metal concentrations of children ages 6-11 surveyed in NHANES.

Talk to your family doctor about health concerns related to metals exposure.

Speaking with your primary doctor or your local health care center is a good starting point in understanding health consequences of metals exposure. Your doctor may be able to address your concerns, or he or she may refer you to a board-certified physician who specializes in medical toxicology or occupational and environmental medicine. While study investigators at the University of Arizona are available to share general knowledge about metals exposures and possible health effects, your primary doctor is the best person to address specific health questions.

Urine was not analyzed at a certified clinical laboratory.

MESH is a university project aimed at understanding exposure to metals; therefore, we chose to analyze urine samples at University of Arizona laboratory facilities. However, these facilities are not certified as clinical laboratories. The analysis procedures and results are checked for quality according to research criteria, not clinical criteria. Only certified clinical laboratories can provide analyses and results on which healthcare decisions can be based. For more information about certified clinical laboratories please visit the website in the Additional Information section.

Understanding Your Results

Your results are divided by type of sample (e.g., water, soil, urine). Each result graphic shows test results for detected metal contaminants.

Important Terms

Here's a set of terms you'll see throughout your results.

- **Concentration** – The amount of metal in a given mass or volume of sample. Sample types include water, soil, dust, urine, and toenails.
- **Creatinine Corrected Concentration** – The concentration of each metal in your urine after adjusting for variations in a person's hydration status by measuring a component in urine called creatinine.
- **Maximum Contaminant Level** – The maximum amount of a regulated metal allowed in drinking water. This level is set by the U.S. Environmental Protection Agency.
- **µg/g** (Micrograms per Gram) – A measure of how many micrograms of a substance (such as metal) are in a gram of a solid, such as toenails.
- **µg/g Creatinine** (Micrograms per Gram Creatinine) – A measure of how many micrograms of a metal are in a volume of urine that contains one gram of creatinine (see above, Creatinine Corrected Concentration). This is a way to measure metals in urine while adjusting for different levels of dehydration people can experience at the time their urine sample is collected.
- **mg/L** (Milligrams per Liter) – A measure of how many milligrams of a substance (such as metal) are in a liter of liquid, such as water.
- **mg/kg** (Milligrams per Kilogram) – A measure of how many milligrams of a substance (such as metal) are are in a kilogram of a solid, such as soil or dust.
- **ND** (None Detected) – Metals with a level of 'ND' have a concentration below what our laboratory can measure or detect.
- **NHANES** (National Health and Nutrition Examination Survey) – A study that periodically assesses the environmental exposures, health, and nutritional status of adults and children in the United States using a wide range of measures.

- **NHANES 50th Percentile** - Fifty percent (50%) of all samples from NHANES participants aged 6-11 years in 2009-2010 are less than this concentration level.

- **NHANES 95th Percentile** – Ninety-five percent (50%) of all samples from NHANES participants aged 6-11 years in 2009-2010 are less than this concentration level.

- **ADEQ Soil Remediation Level** - A level of metal in soil in a residential area that may trigger further study or investigation, but does not necessarily warrant cleanup. This level is determined by the Arizona Department of Environmental Quality.

Metals in Your Water

Legend

- Your Kitchen Tap Water (green bar)
- Your Kitchen Tap Water Resampled on 10/31/12 (purple bar)
- Maximum Contaminant Level (red box)

Note: There are no Maximum Contaminant Levels for aluminum or nickel.

Aluminum

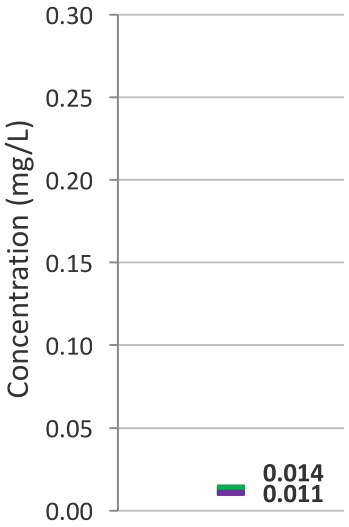

Arsenic

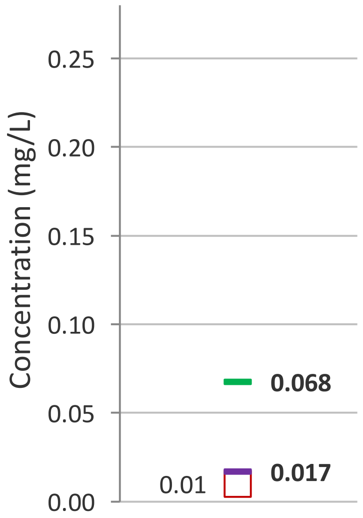

Beryllium

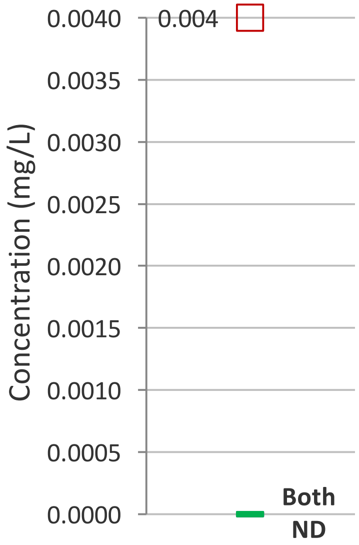

Cadmium

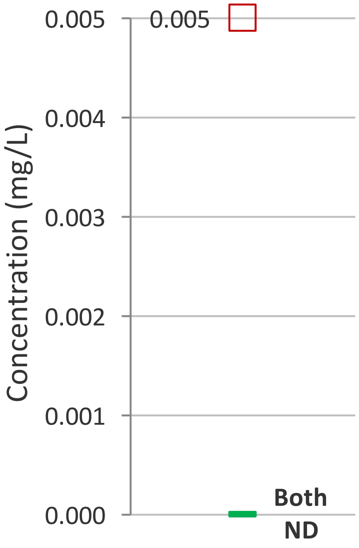

Chromium

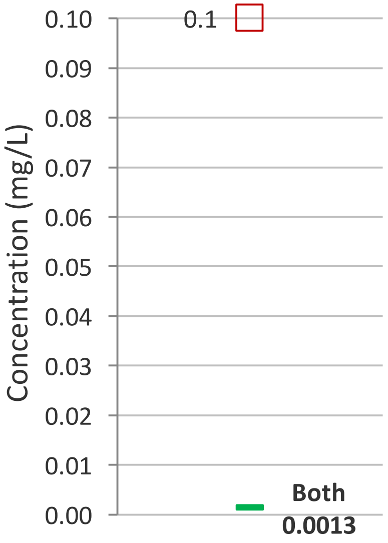

Lead

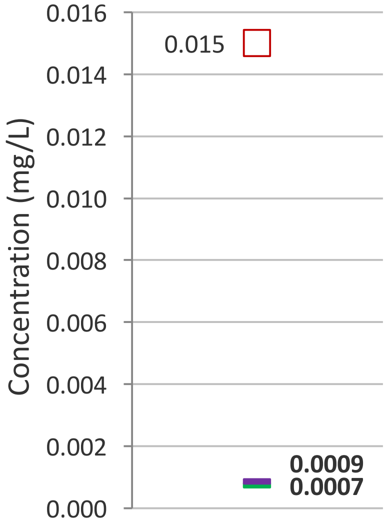

Nickel

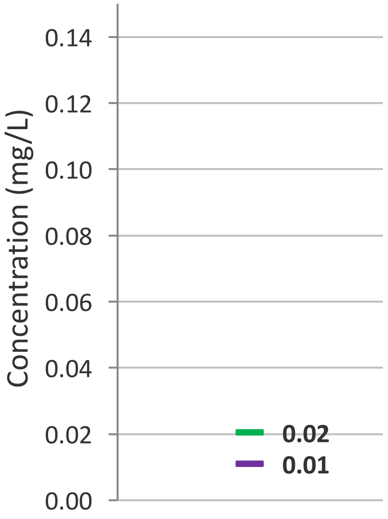

Metals in Your Yard Soil

Legend

- Your Yard Soil
- ADEQ Soil Remediation Level

Aluminum

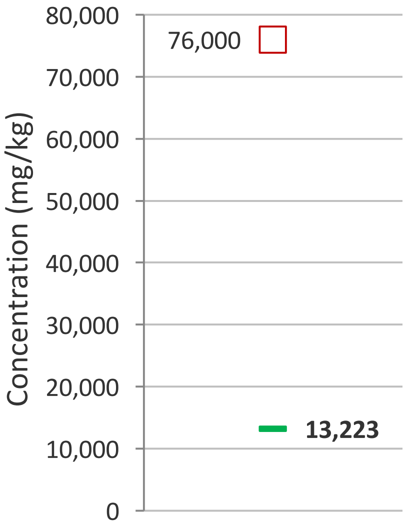

Arsenic

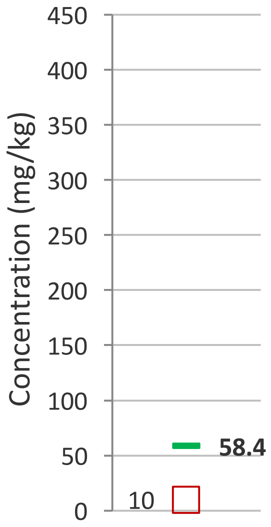

Beryllium

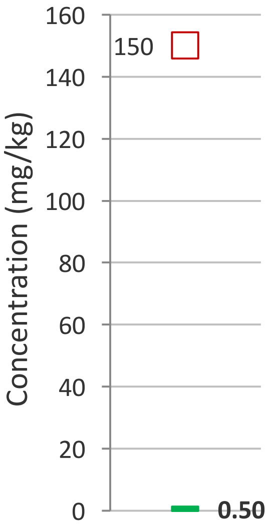

Cadmium

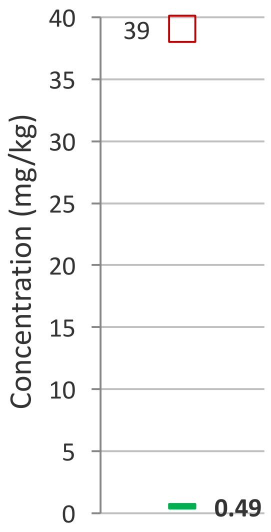

Chromium

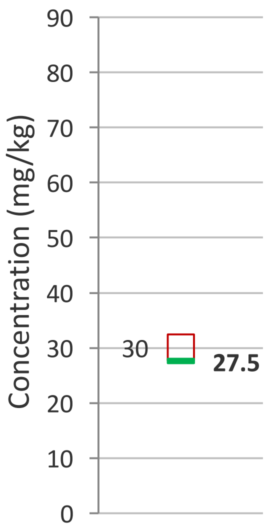

Lead

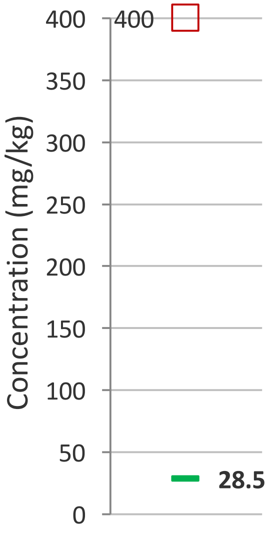

Nickel

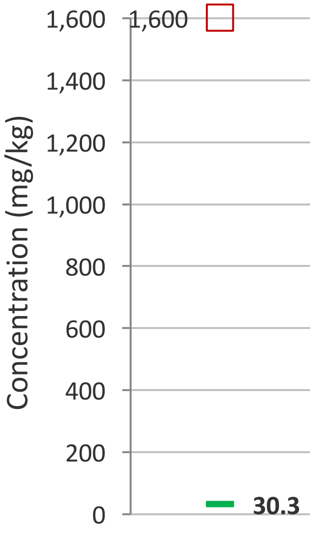

Metals in Your Vacuum Dust

Legend

- Your Vacuum Dust (green bar)
- ADEQ Soil Remediation Level (red square)

Note: There are no guidelines for contaminants in house dust, so we have included soil guidelines for comparison.

Aluminum

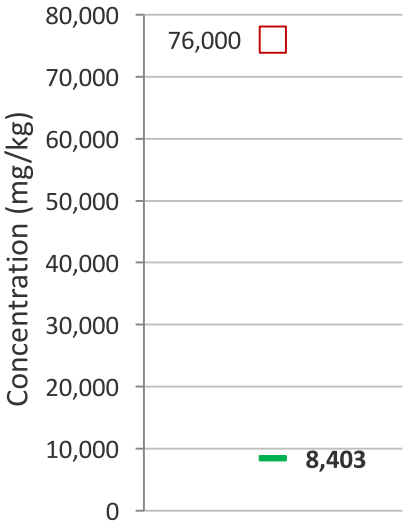

Arsenic

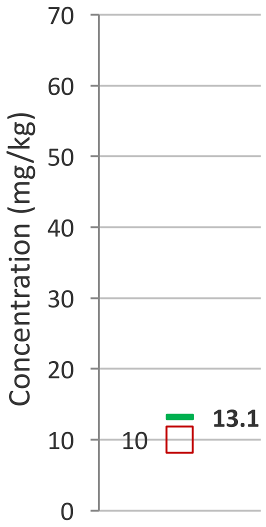

Beryllium

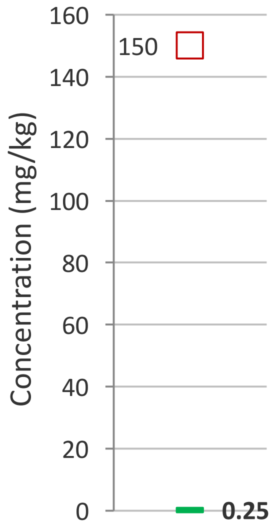

Cadmium

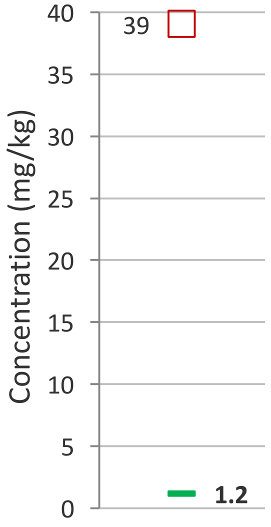

Chromium

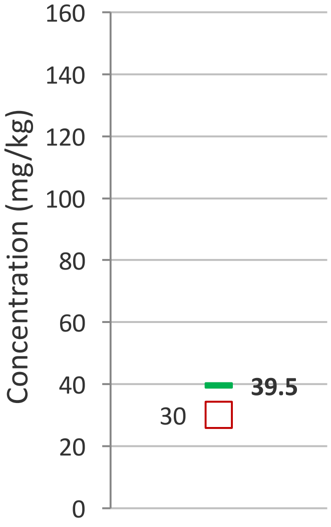

Lead

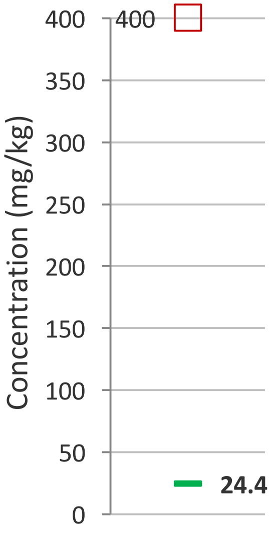

Nickel

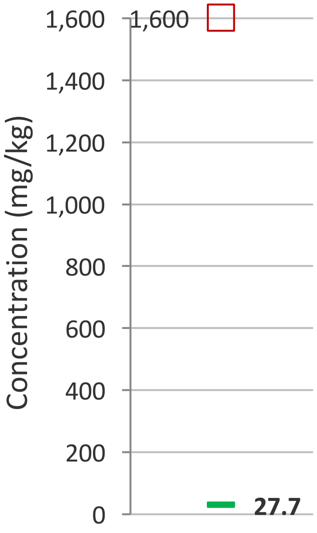

Metals in Urine

Legend

(green bar)	**Child Born on 08/09/10**
(purple bar)	**Child Born on 09/10/11**
△	NHANES 50th Percentile (Creatinine Corrected Concentration)
○	NHANES 95th Percentile (Creatinine Corrected Concentration)

Note: The NHANES values for Cadmium are below what our laboratory can measure. There are no NHANES values for aluminum, beryllium, chromium, and nickel.

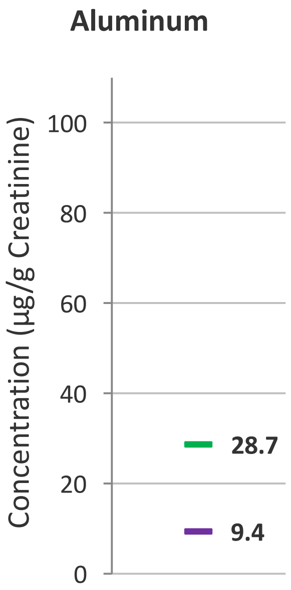

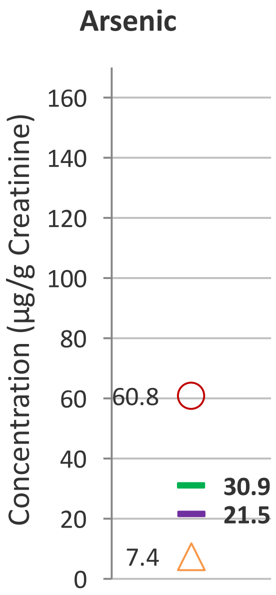

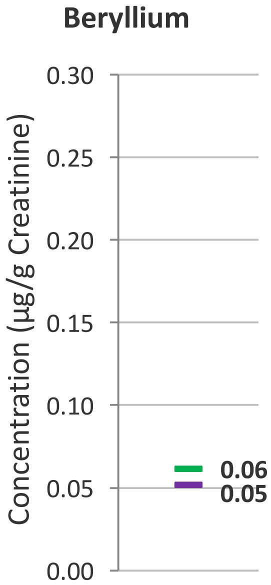

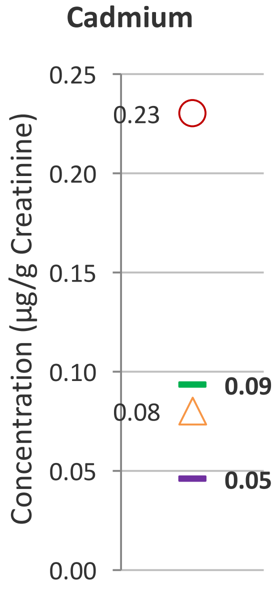

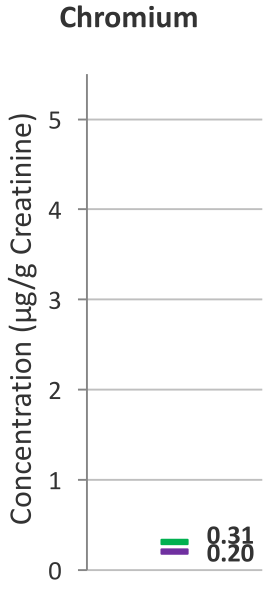

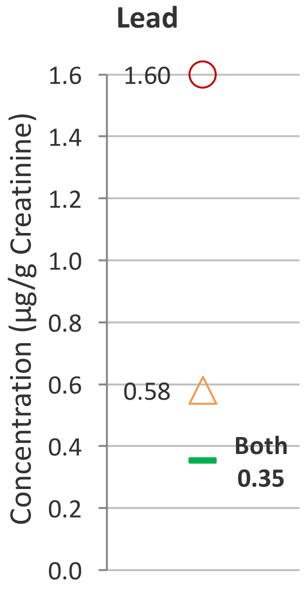

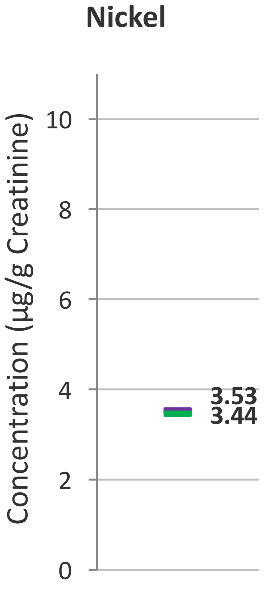

Metals in Toenails

Legend

- Child Born on 08/09/10 (green)
- Child Born on 09/10/11 (purple)

Note: There are no guidelines for metals in toenails.

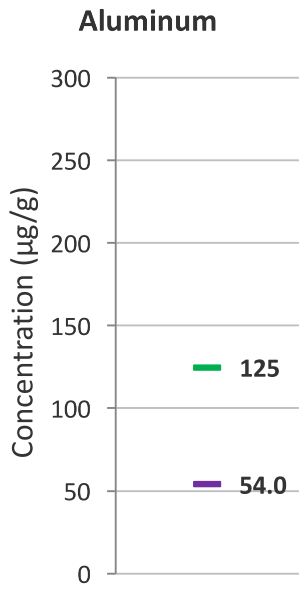

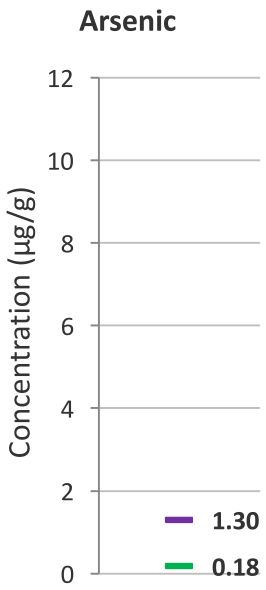

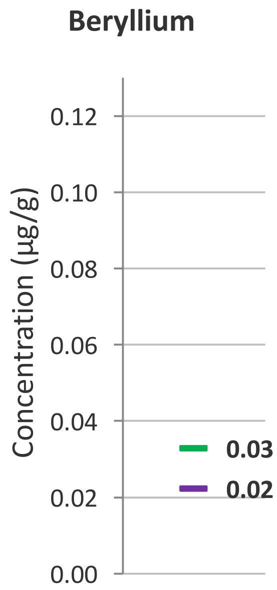

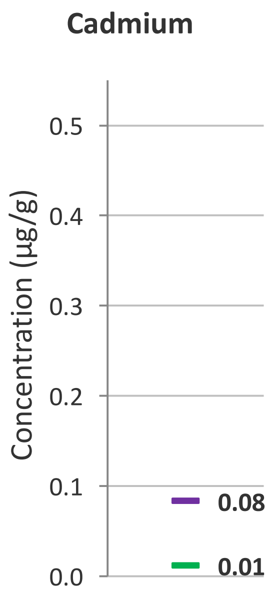

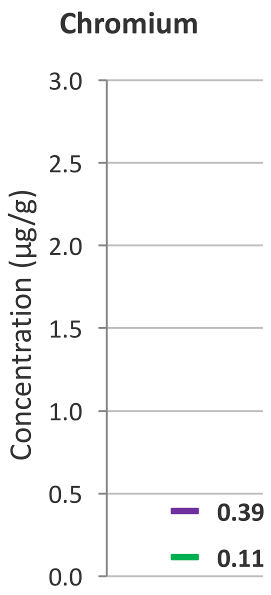

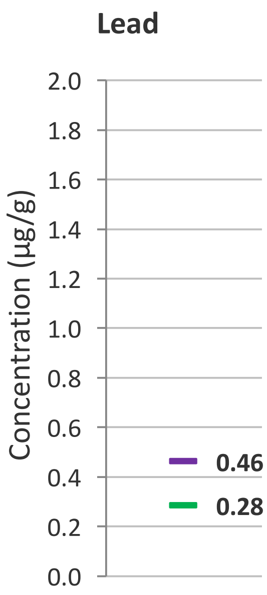

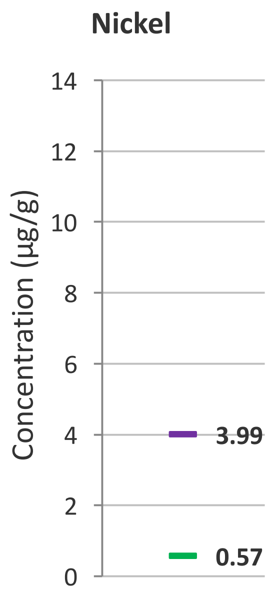

Results References

Please visit these websites listed by subject to find information used in this results packet.

- **ADEQ Soil Remediation Level**
  - Please visit www.azsos.gov/public_services/title_18/18-07.htm and find the table underneath the section R18-7-210 - Notice of Remediation and Repository. Values are in the "Residential (mg/kg)" column.
- **Creatinine Corrected Concentration**
  - Please visit www.cdc.gov/exposurereport/pdf/FourthReport.pdf and scroll down to page five (5). The definition is under the "Units" subheading.
- **Maximum Contaminant Level**
  - Please visit www.water.epa.gov/drink/contaminants/index.cfm and scroll down to the "Inorganic Chemicals" heading. Values are in the "MCL or TT" column.
- **NHANES 50th Percentile** and **NHANES 95th Percentile**
  - Please visit www.cdc.gov/exposurereport/pdf/FourthReport_UpdatedTables_Mar2013.pdf and browse to the "Metals and Metalloids" section. Find charts that read, "Urinary *Metal* (creatinine corrected)" in the top left corner. For example, "Urinary Total Arsenic (creatinine corrected)". In the first column, navigate to the "Age Group – 6-11 years"; this relates to the ages of children sampled. Next, move to the second column and find the row "09-10" for "6-11 years"; this denotes the years kids 6-11 years were sampled: 2009-2010. See values in this row that fall in the "50th" and "95th" percentiles columns.

Additional Information

Please visit these websites listed by subject to find additional information.

- **Agency for Toxic Substances and Disease Registry Toxicological Profiles**
  - www.atsdr.cdc.gov/toxprofiles/index.asp
- **American Academy of Pediatrics – Healthy Children**
  - www.healthychildren.org
- **Arizona Department of Environmental Quality**
  - www.azdeq.gov
- **Arizona Department of Health Services**
  - www.azdhs.gov
- **Clinical Laboratory Improvement Amendments**
  - www.cms.gov/Regulations-and-Guidance/Legislation/CLIA/index.html?redirect=/clia
- **Environmental Protection Agency**
  - www.epa.gov
- **NHANES** (National Health and Nutrition Examination Survey)
  - www.cdc.gov/exposurereport/pdf/FourthReport.pdf

MESH

Metals Exposure Study in Homes

Summary
of Study Results

Superfund Research Program

Superfund Research Program
The University of Arizona

The University of Arizona

Friday, June 17, 2016

Dear Participant,

Thank you again for participating in the University of Arizona (UA) Metals Exposure Study in Homes (MESH). This packet shows the test results of your children's urine, toenails, and blood and your home's drinking water, yard soil, and vacuum dust compared to those of other MESH participant households. All of these samples have been tested for seven metals: aluminum, arsenic, beryllium, cadmium, chromium, lead, and nickel, with the exception of your children's blood, which was tested for only lead. This packet is the second of two results packets. If you did not receive your first packet, please contact Nathan Lothrop toll-free at (877) 535-6171.

We are trying to understand how much you and your family are exposed to metals. We are also trying to understand how metals can enter the home. Results from this study are helping us determine how people can reduce their exposure to metal contaminants; however, we are still analyzing study results to find additional ways you can reduce your exposure to metals.

In this packet, we have included the following:

- a summary of the study results;
- ways you can reduce your exposure to metals;
- important information about MESH;
- a glossary of terms that describes information you will find in your results; and
- additional information on the environmental and health guidelines.

Please keep in mind that the methods used in this study were designed for research purposes only and do not necessarily follow US Environmental Protection Agency or Clinical Laboratory Improvement Amendments standard methods.

If you have questions, please contact Nathan Lothrop toll-free at (877) 535-6171 or lothrop@email.arizona.edu. Thank you again for participating; you have helped us better understand metals exposures and how to reduce them.

Sincerely,

Walter T. Klimecki. DVM, Ph.D.
Associate Professor of Pharmacology and Toxicology
University of Arizona

Study Summary

BACKGROUND

Thank you again for participating in the Metals Exposure Study in Homes (MESH); we could not have done this without you! MESH began in 2012 in response to Dewey-Humboldt community concerns about whether contamination from the Iron King Mine and Humboldt Smelter Superfund Site (the Superfund Site) was entering people's bodies at harmful levels. We investigated if levels of metals found in the environment and in people's bodies in Dewey-Humboldt are higher than environmental and health guidelines. Altogether, 70 children 1-11 years of age from 34 homes in the Dewey-Humboldt area participated in MESH (Figure 1).

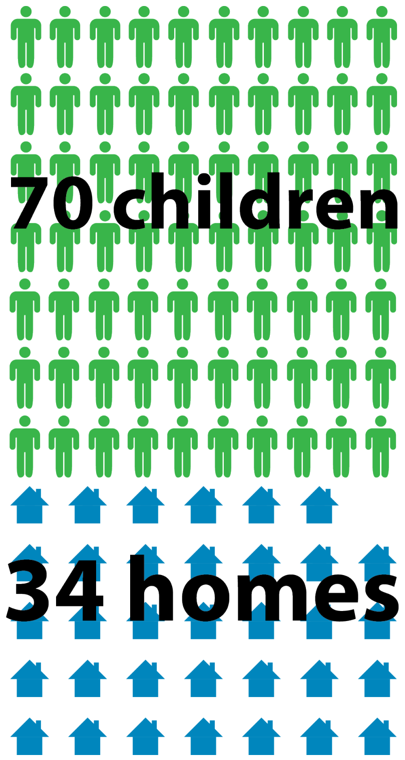

Figure 1: 70 children ages 1-11 years from 34 homes participated in MESH.

We analyzed children's urine, toenails, and blood, as well as drinking water, yard soil, and vacuum dust from their homes. Samples were tested for seven metals: aluminum, arsenic, beryllium, cadmium, chromium, lead, and nickel, with the exception of blood, which was tested only for lead. We focused on these seven metals because the US Environmental Protection Agency (EPA) noted them as metals of potential concern based on air and soil samples taken on or near the Superfund Site in its 2010 Remediation Investigation of the Superfund Site. For more information about this report and other EPA work at to the Superfund Site, please visit the EPA website in the Additional Information section.

We focused on children for two reasons: 1) children are still growing and may be more susceptible to health effects of high exposures to pollutants; and 2) children are more likely than adults to put their hands or objects like toys into their mouths, which may be covered with soil and dust. It is important to know that the metals we tested occur naturally in the environment, including in Arizona, and can also come from man-made sources, such as lead in older paint. So, even if the Superfund Site were not present, residents could still be exposed to these metals from other sources.

RESULTS

Overall, we found that levels of most metals were below the Maximum Contaminant Level (MCL) in drinking water and below the Arizona Department of Environmental Quality Soil Remediation Level (AZ SRL) in yard soil and vacuum dust in MESH homes. However, arsenic levels in drinking water, yard soil, and vacuum dust were above these respective guidelines for many MESH homes. In addition, we found most metals levels in urine for most children in MESH were below those of a typical child in the US. However, arsenic levels in urine for most children in MESH were higher than a typical child in the US.

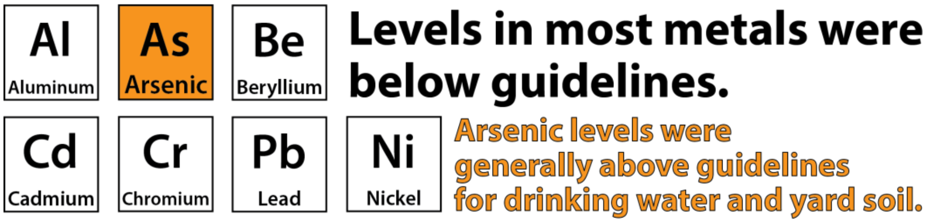

Figure 2: Seven metals were analyzed in MESH; we believe Dewey-Humboldt residents have higher arsenic exposure compared to typical US residents.

Based on these results, **we believe that MESH participants have higher levels of exposures to arsenic compared to typical US residents** (Figure 2). Concentrations of all seven metals in drinking water, yard soil, vacuum dust, urine, and toenails and lead in blood, for your household and for other MESH households, are found in later sections. In the summary sections that begin on the next page, we only focus on arsenic in urine, drinking water, yard soil, and vacuum dust and lead in blood. Toenails are not included in the summarized results because there is no guideline for comparison.

REDUCING YOUR EXPOSURE TO ARSENIC

During the study, we tried to speak with a parent or guardian about their household's results and how they compared to guidelines. If you were not called about your results and would like to discuss them, please call Nathan Lothrop toll-free at (877) 535-6171.

For suggestions on how to reduce your exposure to arsenic, please read the following summarized results sections and the included factsheets, "Arsenic in drinking water: what you need to know" and "How to Reduce Your Exposure to Arsenic and Lead in Dewey-Humboldt, Arizona." If you still have questions or concerns, please call Nathan Lothrop toll-free at (877) 535-6171.

Urine

INTRODUCTION

Arsenic and other metals found in urine can come from many sources. MESH was designed to estimate your potential exposure from common sources of these metals, such as drinking water, yard soil, and house dust (measured in vacuum dust). We did this by taking samples from your home environment and your children, and with questionnaires and food and activity diaries. Arsenic is also found in certain foods; however, we were unable to sample any food. For information about arsenic in food (including drinks), please visit the Food and Drug Administration website found in the Additional Information section.

RESULTS

Overall, most metals were below the median level of children in a national study (the National Health and Nutrition Examination Survey, NHANES) conducted regularly by the US Centers for Disease Control and Prevention (CDC) (Figure 3). This median level is also called the NHANES 50th percentile, which means 50% (half) of children's values in NHANES are below this value, and 50% (half) are above it. However, the arsenic level in urine was above the NHANES 50th percentile for 56 of 68 children (82%) in MESH, compared to a group of 68 typical children, in which only 34 of 68 children (50%) would have arsenic levels in urine above the NHANES 50th percentile.

However, arsenic levels in urine of MESH participants are generally similar to those of other Arizona residents of all ages.

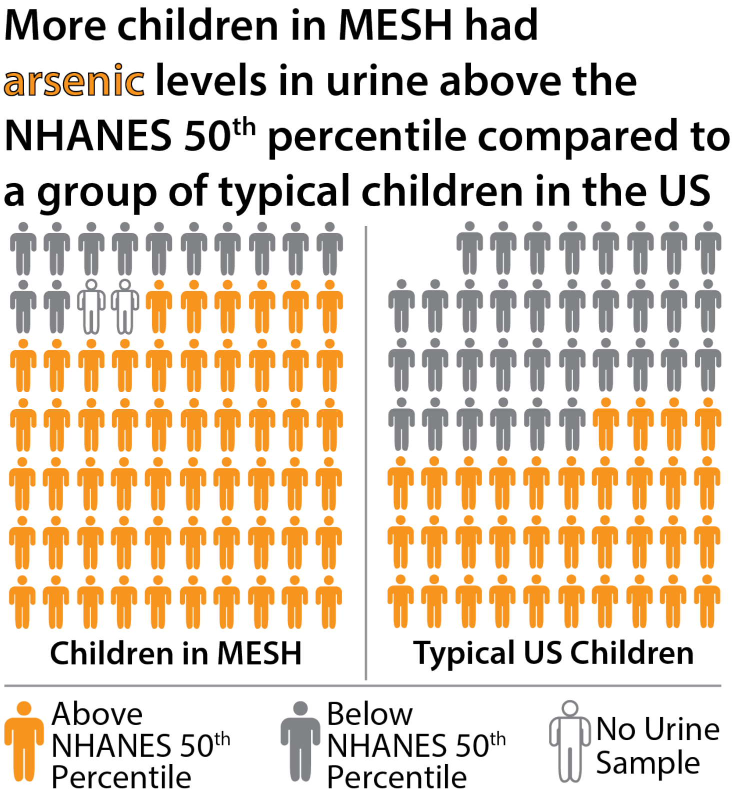

Figure 3: The arsenic level in urine was above the NHANES 50th percentile for 56 of 68 children (82%) in MESH, compared to a group of 68 typical children, in which only 34 of 68 children (50%) would have arsenic levels in urine above the NHANES 50th percentile.

Based on these results in urine, we believe that MESH participants have higher exposures to arsenic compared to typical US residents. At the same time, these results are similar to those that have been measured in other Arizona residents of all ages.

INTERPRETING ARSENIC LEVELS IN YOUR CHILD'S URINE

Currently, there is no health standard or guideline for arsenic in urine for children. Given this, if you are concerned about your child's urinary arsenic level, start reducing their potential exposure to arsenic from drinking water, yard soil, and house dust using tips from the following sections and in the included factsheets, "Arsenic in drinking water: what you need to know" and "How to Reduce Your Exposure to Arsenic and Lead in Dewey-Humboldt, Arizona." Additionally, it is important to remember that urine shows how much of a metal was absorbed by the body in the previous 3-4 days. As a result, a single urine test may not give an accurate picture of past (or future) exposures.

Finally, ask your family doctor for help understanding possible health risks from exposure to arsenic. **If your family doctor is unsure how to interpret your child's urine results,** please have him or her call Nathan Lothrop at (877) 535-6171 for referral to a board-certified physician who specializes in medical toxicology or occupational and environmental medicine.

Blood

RESULTS

None of the 46 children (0%) in MESH who had their blood tested for lead had levels above the reference value of 5 µg/dL set by the CDC (Figure 4). Based on this, **we do not believe lead exposure is a particular concern for residents in and around Dewey-Humboldt**. The Arizona Department of Health Services recommends all children in Arizona have their blood tested for lead at age 1, and again at age 2. If you think your child of any age has come in contact with lead, test that child's blood for lead.

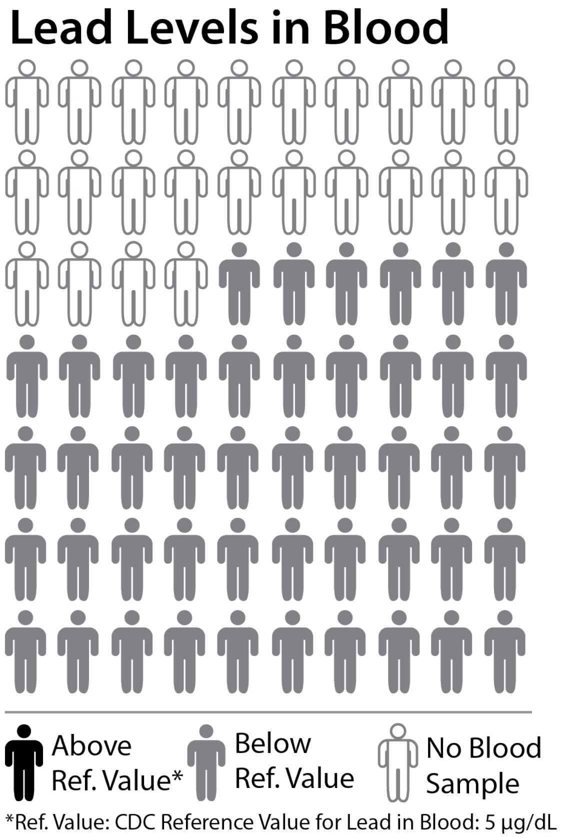

Figure 4: None of the 46 children (0%) in MESH who had their blood tested for lead had lead levels above the reference value set by the CDC.

Drinking Water

RESULTS

While levels of most metals in drinking water were below the Maximum Contaminant Level (MCL) set by the EPA, arsenic levels in drinking water were above the MCL in 19 of 33 (56%) homes in MESH (Figure 5). In this case, drinking water is not treated by an in-home reverse osmosis system, which can reduce arsenic levels in water. The MCL is the maximum amount of a contaminant allowed in drinking water so that it is still safe to drink over many years. The MCL for arsenic is 10 parts per billion (ppb); this may also be written as 10 micrograms per liter (µg/L).

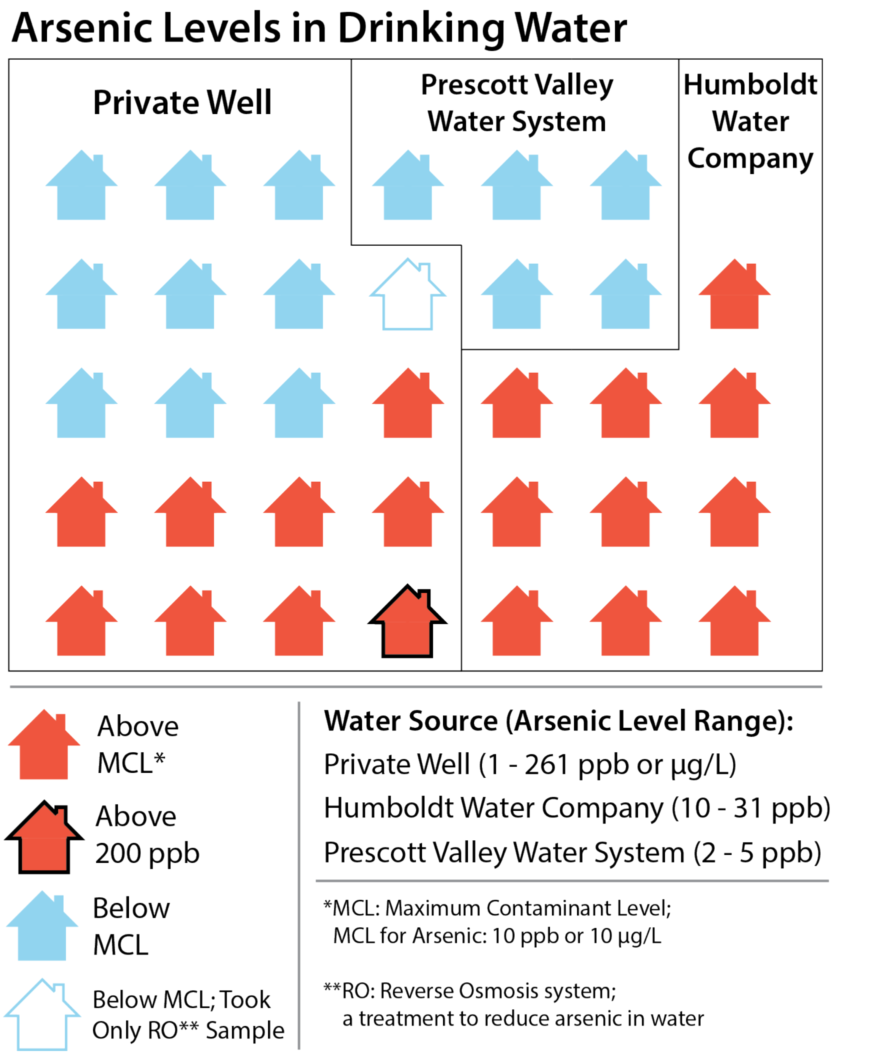

Figure 5: The arsenic level in drinking water was above the MCL in 19 of 33 homes (56%) in MESH.

We found the highest arsenic levels in private wells, with the highest value being 26 times that of the MCL. However, this does not mean that all private wells had arsenic levels above the MCL. In addition, some homes using Humboldt Water Company water had arsenic levels above the MCL, with the highest value being 3 times that of the MCL. Humboldt Water Company is aware of this issue and is currently installing a treatment system to reduce the arsenic in their water.

ARSENIC IN DRINKING WATER: WHAT YOU NEED TO KNOW

High arsenic levels are present in groundwater in the Dewey-Humboldt area. Please refer to the enclosed factsheet, “Arsenic in drinking water: what you need to know,” for information and resources on reducing arsenic in drinking water. Further, the Arizona Department of Health

Services advises the following actions if the arsenic level in your water is above the MCL of 10 parts per billion (ppb):

- *If the arsenic level in your water is between* ***10 – 200 ppb***, it is not an emergency, but make plans to get water for drinking and cooking from a different source, or install and maintain a reverse osmosis home treatment system.
- *If the arsenic level in your water is* ***over 200 ppb***, DO NOT use this water for drinking or cooking. Use another water source immediately until you can install and maintain a home treatment system. If the arsenic level in your water is less than 500 ppb, you can continue to use this water for bathing and washing clothes and dishes.

If you use a private well, test your water at least once every three years to determine the amount of arsenic and other metals in your water.

Yard Soil and Vacuum Dust

INTRODUCTION

We used the Arizona Department of Environmental Quality Soil Remediation Level (AZ SRL) as a reference for metal concentrations in yard soil and vacuum dust. We are comparing vacuum dust levels to the AZ SRL since there are no guidelines for vacuum dust. The AZ SRL is a yard soil screening level based on a health risk assessment not specific to conditions in Dewey-Humboldt. Metal **levels in soil higher than the AZ SRL do not necessarily require cleanup**. The EPA, the Agency for Toxic Substances and Disease Registry (ATSDR), and the Arizona Department of Health Services (ADHS) are currently investigating soils in this area to determine if there is a health risk to residents from the metals in local soils. Dewey-Humboldt residents should refer to the results of the EPA, ATSDR, and ADHS study for guidance on whether their yard soil poses health risks and if it will be cleaned up. Until these results are available, preventing outdoor soil from coming into your home is one way of reducing arsenic levels in your vacuum dust.

RESULTS

Levels of most metals in yard soil and vacuum dust (previously referred to as Personal Vacuum Bag Dust) were below the AZ SRL. Arsenic levels were above the AZ SRL in yard soil for 27 of 34 homes (79%) in MESH (Figure 6). Arsenic levels in vacuum dust were above the AZ SRL in 17 of 31 homes (55%) (Figure 7).

REDUCING YOUR EXPOSURE TO YARD SOIL AND VACUUM DUST WITH HIGH ARSENIC LEVELS

Frequent vacuuming with a HEPA filter, wet dusting and mopping, and not wearing shoes indoors are ways of reducing the amount of dust that come into your home from outside. For more information on reducing your exposure to outdoor dust in your home, please refer to the enclosed factsheet “How to Reduce Your Exposure to Arsenic and Lead in Dewey-Humboldt, Arizona.”

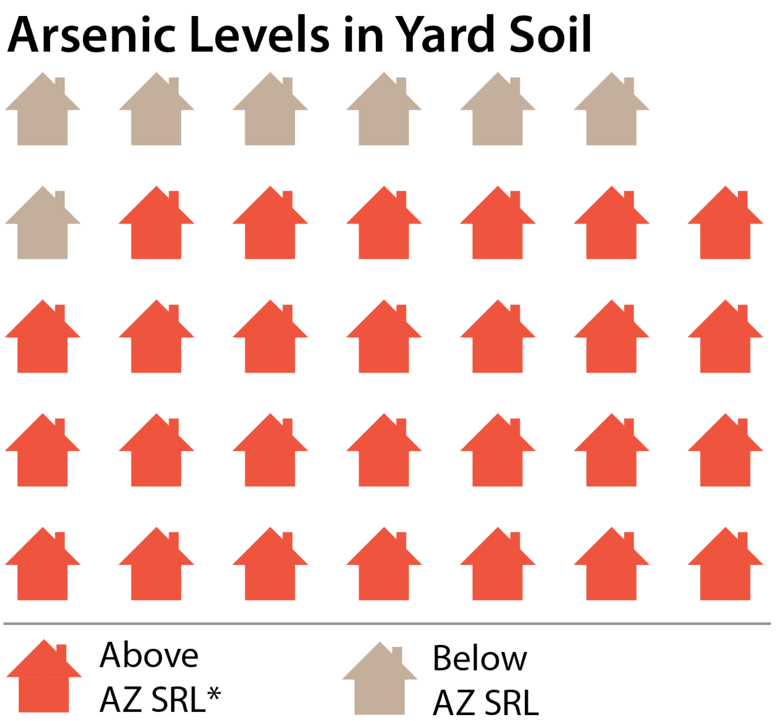

*AZ SRL: AZ Department of Environmental Quality Soil Remediation Level: AZ SRL for Arsenic: 10 ppm or 10 mg/kg

Figure 6: The arsenic level in yard soil was above the AZ SRL in 27 of 34 homes (79%) in MESH.

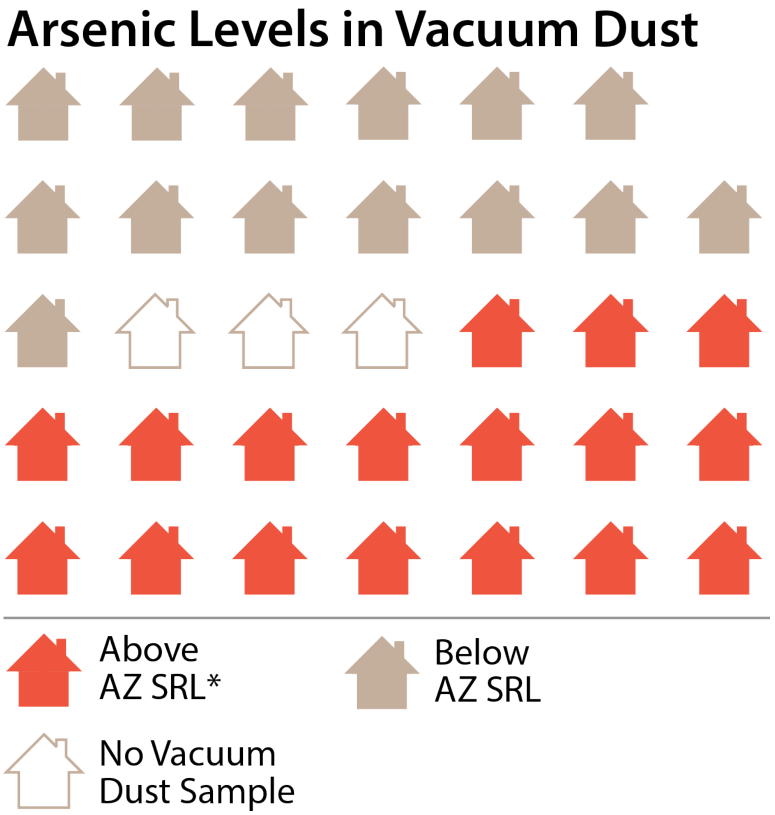

*AZ SRL: AZ Department of Environmental Quality Soil Remediation Level: AZ SRL for Arsenic: 10 ppm or 10 mg/kg

Figure 7: The arsenic level in vacuum dust was above the AZ SRL in 17 of 31 homes (55%) in MESH.

Exposure to Arsenic and Health Effects

The findings of MESH (higher levels of arsenic in urine, drinking water, yard soil, and vacuum dust) do not mean that MESH participants will have health problems from exposure to arsenic. We cannot determine whether a person's current health problems are due to his or her exposure or whether a person will develop future health problems. Researchers are still studying the relationship between levels of arsenic in the body and various diseases.

> MESH findings **do not mean** Dewey-Humboldt residents will have health problems from exposure to arsenic.

If arsenic levels in your water are above the Maximum Contaminant Level (MCL), we recommend reducing your exposure with methods outlined in the enclosed factsheet, "Arsenic in drinking water: what you need to know." If arsenic levels in your yard soil or vacuum dust are above the Arizona Department of Environmental Quality Soil Remediation Level (AZ SRL), we recommend reducing your exposure with methods outlined in the enclosed factsheet, "How to Reduce Your Exposure to Arsenic and Lead in Dewey-Humboldt, Arizona." In addition, your family doctor may be able to help you understand possible health risks from exposure to levels of metals above guidelines.

If your family doctor is unsure how to interpret your child's urine results, please have him or her call Nathan Lothrop toll-free at (877) 535-6171 for referral to a board-certified physician who specializes in medical toxicology or occupational and environmental medicine.

If you have any questions or concerns regarding:

- …**results in this packet**, please contact Nathan Lothrop toll-free at (877) 535-6171 or at lothrop@email.arizona.edu.
- …**metals exposures and health**, please contact Jennifer Botsford, an environmental health expert familiar with MESH, at the Arizona Department of Health Services at (602) 364-3128.
- …**the Iron King Mine and Humboldt Smelter Superfund Site**, please contact Amanda Pease, the EPA Community Involvement Coordinator for the Superfund Site, toll-free at (800) 231-3075 or at (415) 972-3068.

Important Information about MESH

MESH is an exposure study, not an epidemiology study.

An epidemiology study attempts to explain the relationship between an exposure and a health outcome in a defined group of people. For example, large-scale epidemiology studies established that smoking cigarettes (an exposure) increases the risk of lung cancer (a health outcome). An exposure study is designed to determine and understand the contact a person has with an environmental factor. MESH is an exposure study designed to find out if people are coming into contact with metals through drinking water, yard soil, or house dust and how much exposure they may be getting. Though MESH did collect some health information in a questionnaire, this was done to see if health outcomes should be assessed in a future study. At the moment, we are still analyzing data to determine if any health outcomes should be evaluated in the future.

MESH is designed to help us understand which factors can contribute to increased metals exposures.

This study is investigating housing characteristics, behavior routines, general demographic information, and several other factors to see how these may impact the levels of metals in your home. We will use this information to provide additional suggestions to participants and other community members about how they can reduce their exposure to metals. However, we have not yet completed these more complex analyses. Nevertheless, there are many effective ways to reduce your exposure to metals, as outlined in this packet and in the included factsheets.

Metals occur naturally in the environment.

Some areas have naturally higher levels of metals than others (which explains why mines are usually developed in such areas with higher levels of metals). Furthermore, while we can measure the level of a metal in certain substances like water or soil, it is difficult to know the original source of the metal. For example, arsenic may be found in soil in many parts of Arizona simply because it naturally occurs there. Similarly, some drinking water wells may have high arsenic concentrations because rock touching the well water has arsenic in it. Arsenic can also be found in some foods and drinks and in pressure-treated wood manufactured before 2004. One objective of MESH is to determine if living closer to the Superfund Site is associated with higher levels of exposure to certain metals for residents in and around Dewey-Humboldt. However, we have not yet completed these more complex analyses. As a result, we do not yet know if, and to what extent, the Superfund Site contributes to metals exposure in people.

Single exposure measurements are "snapshots in time."

We measured metal concentrations at a single point in time. Measuring metal concentrations with a single sampling does not establish what the exposures were in the past or what they may be in the future. Some exposure measurements can only reflect exposure over very short time periods. For example, arsenic testing in urine only reflects arsenic exposure over the past several days. Likewise, metal concentrations in house dust may change from week to week, depending on the dust sources and the frequency of cleaning. As a result, measurements taken only once may not give an accurate picture of past (or future) exposures. This is important, because many chronic diseases associated with environmental exposures only happen after long periods of exposure.

Where available, we have used NHANES measurements as points of reference for urine metal concentrations.

The National Health and Nutrition Examination Survey (NHANES), is a US Centers for Disease Control and Prevention program that annually examines about 5,000 people as young as 6 years of age across the United States to assess environmental exposures, health, and nutrition status. Where NHANES data is available for metal concentrations in urine, we have provided reference values for the 50th and 95th percentile of the children surveyed. The 50th percentile value means that half the children between ages 6 and 11 surveyed had urine metal measurements below this value and half were above that value. The 95th percentile value means that only 5% of the children had urine metal concentrations higher than that value. These percentile values are not health standards or guidelines; they simply allow us to compare our results to the results of children ages 6-11 from across the nation, surveyed in NHANES. In MESH, we also enrolled children ages 1-5, but NHANES does not have urine metal concentrations for this age range.

Talk to your family doctor about health concerns related to metals exposure.

Speaking with your primary doctor or your local health care center is a good starting point in understanding health risks of metals exposure. While study investigators at the University of Arizona are available to share general knowledge about metals exposures and possible health effects, your primary doctor is the best person to address specific health questions. **If your family doctor is unsure how to interpret your child's urine results**, please have him or her call Nathan Lothrop toll-free at (877) 535-6171 for referral to a board-certified physician who specializes in medical toxicology or occupational and environmental medicine.

Urine, drinking water, yard soil, and vacuum dust were analyzed at University of Arizona laboratory facilities.

MESH is a university project aimed at understanding exposure to metals; therefore, we chose to analyze urine, drinking water, yard soil, and vacuum dust samples at University of Arizona laboratory facilities. Although these facilities are not certified as clinical laboratories, our procedures and results undergo rigorous quality control testing according to research criteria. Our methods differ from clinical or EPA methods because they are designed for research purposes, and cannot serve as the basis for healthcare decisions or environmental cleanup action. For more information about certified clinical laboratories, please visit the Clinical Laboratory Improvement Amendments website found in the Additional Information section.

Understanding Your Results

Your results are divided by type of sample (e.g., water, soil, urine). Each result chart shows test results for detected metal contaminants.

Important Terms

Here is a set of terms you will see throughout your results:

- **AZ SRL** (Arizona Department of Environmental Quality Soil Remediation Level) –The AZ SRL is a yard soil screening level based on a health risk assessment not specific to conditions in Dewey-Humboldt. If a metal concentration in residential yard soil is above the AZ SRL, it suggests further study, but does not necessarily require cleanup. This level is set by the Arizona Department of Environmental Quality.

- **Concentration** – The amount of metal in a given volume or mass of sample, such as: µg/L, mg/kg, µg/g, or µg/dL. Sample types include water, yard soil, vacuum dust, urine, toenails, and blood.

- **MCL** (Maximum Contaminant Level) – The MCL is the maximum amount of a contaminant allowed in drinking water so that it is still safe to drink over many years. This level is set by the US Environmental Protection Agency.

- **µg/L** (Micrograms per Liter**)** – A measure of how many micrograms (one-thousandth of a milligram) of a substance (such as a metal) are in a liter of liquid (such as water or urine). This measure is also referred to as parts per billion (ppb). For perspective, 1 µg/L or ppb is the equivalent to a drop of ink in a backyard swimming pool.

- **mg/kg** (Milligrams per Kilogram) – A measure of how many milligrams of a substance (such as a metal) are in a kilogram of a solid (such as yard soil or vacuum dust). This measure is also referred to as parts per million (ppm). For perspective, 1 mg/kg or ppm is the equivalent to one penny in $10,000 in pennies.

- **µg/g** (Micrograms per Gram) – A measure of how many micrograms of a substance (such as a metal) are in a gram of a solid (such as toenails). This measure is also referred to as parts per million (ppm).

- **µg/dL** (Micrograms per Deciliter**)** – A measure of how many micrograms of a substance (such as lead) are in a liter of liquid (such as blood).

- **ND** (None Detected) – Metals with a level of "ND" have a concentration below what our laboratory can measure or detect.

- **NHANES** (National Health and Nutrition Examination Survey) – A study that periodically assesses the environmental exposures, health, and nutritional status of adults and children in the US using a wide range of measures.

- **NHANES 50th Percentile** – Fifty percent (50%) of all samples from NHANES participants aged 6-11 years in 2009-2010 are less than this concentration level, and fifty percent (50%) of all samples are greater than this concentration level.

- **NHANES 95th Percentile** – Ninety-five percent (95%) of all samples from NHANES participants aged 6-11 years in 2009-2010 are less than this concentration level, while only five percent (5%) of all samples are greater than this concentration level.

- **ppb (Parts per Billion)** – A measure of how many units of a substance (such as a metal) are in a billion units of a substance (such as water or urine). For liquids, ppb is the same as µg/L. 1 ppb is the equivalent to a drop of ink in a backyard swimming pool.

- **ppm (Parts per Million)** – A measure of how many units of a substance (such as a metal) are in a million units of a substance (such as yard soil, vacuum dust, or toenails). Ppm is the same as mg/kg or µg/g. 1 ppm is the equivalent to one penny in $10,000.

Interpreting Your Results Charts

In the next section, your results and other MESH participants' results for each of the seven metals we analyzed are illustrated on dot charts, with the exception of blood, which was only analyzed for lead. On the charts, each dot corresponds to one child (charts of urine, toenails, and blood) or one sample from a home (charts of drinking water, yard soil, and vacuum dust). The further to the right a dot is on the chart, the higher the concentration for that dot. Colored dots are children or samples from your own home. Gray dots are children or samples from other MESH homes. Black text in the chart area shows your child's or home's sample concentration for that metal. Colored lines and text in the chart show guidelines or comparison values. If you do not see colored lines or text in the chart, look below the legend for notes on why there are no colored lines or text. On the next page is an example chart that explains the different parts of each chart. If you have questions on any of the charts, please call Nathan Lothrop toll-free at (877) 535-6171.

Example Chart

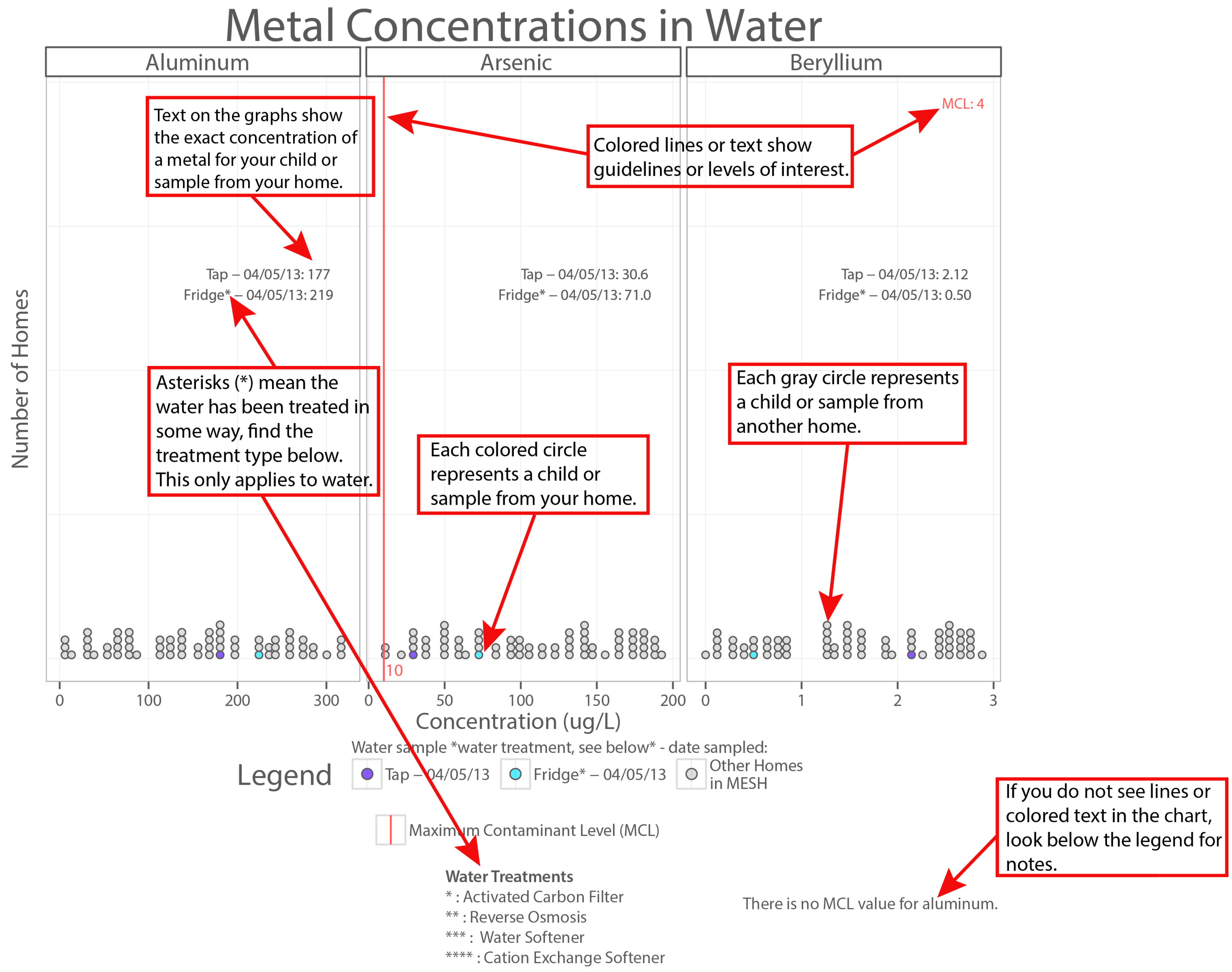

This chart is created from fake data.
It illustrates how the graphs in packets given to participants might look.

Metal Concentrations in Water

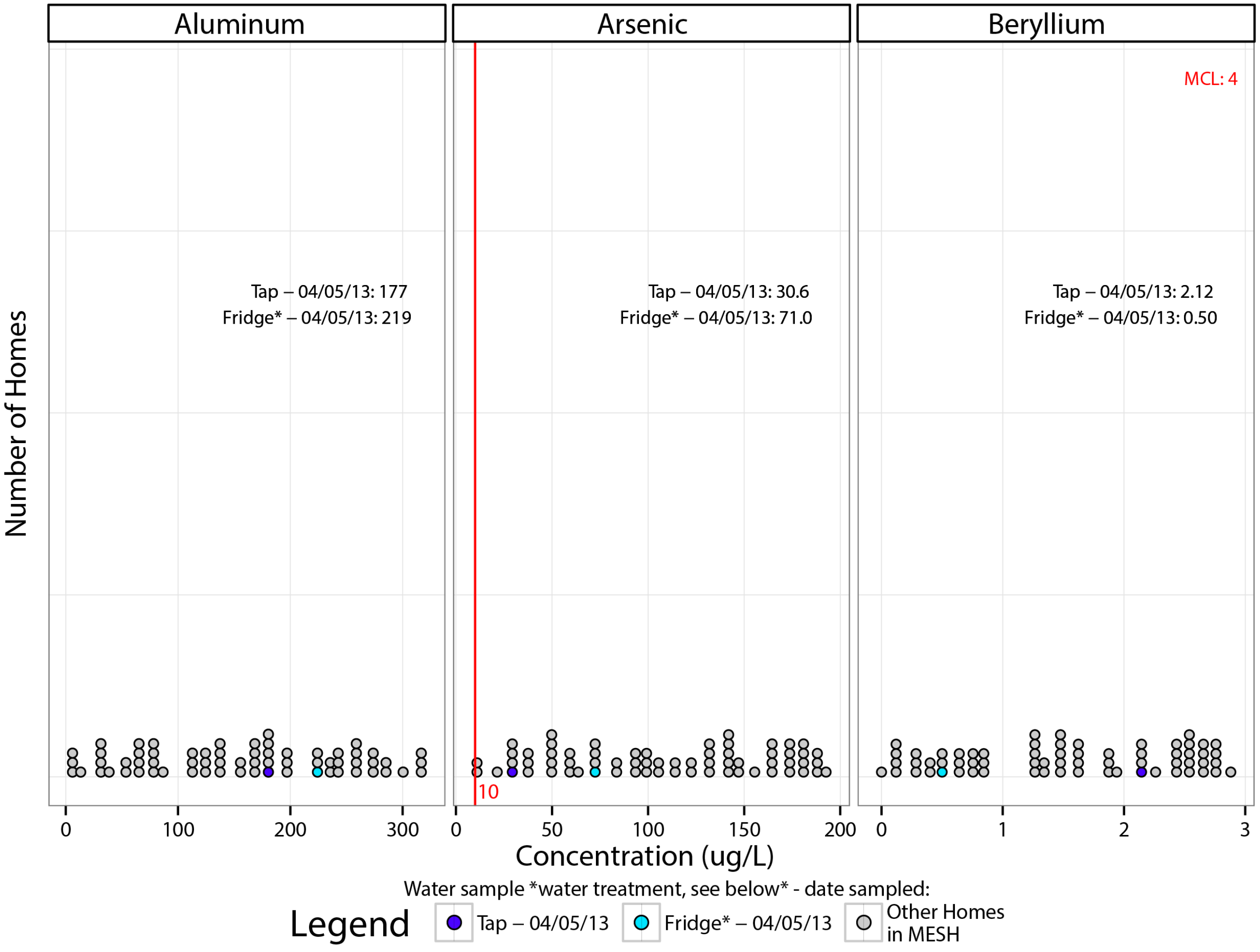

Water samples shown come from many sources, including: private wells (36 samples); Humboldt Water Co. (18); Prescott Valley Water System (8); and bottled water (3).

Water samples also include many types of water treatments, including: activated carbon (7 samples); reverse osmosis (8); water softener (7); cation exchange filter (1); and no treatment (42).

ND (None Detected): Metals with a level of "ND" have a concentration below what our laboratory can measure or detect.

Water Treatments
* : Activated Carbon Filter
** : Reverse Osmosis
*** : Water Softener
**** : Cation Exchange Softener

Note: The dots represent all water samples from homes in MESH. Each dot represents one water sample from a home in MESH.

There is no MCL value for aluminum.

This chart is created from fake data.
It illustrates how the graphs in packets given to participants might look.

Metal Concentrations in Water

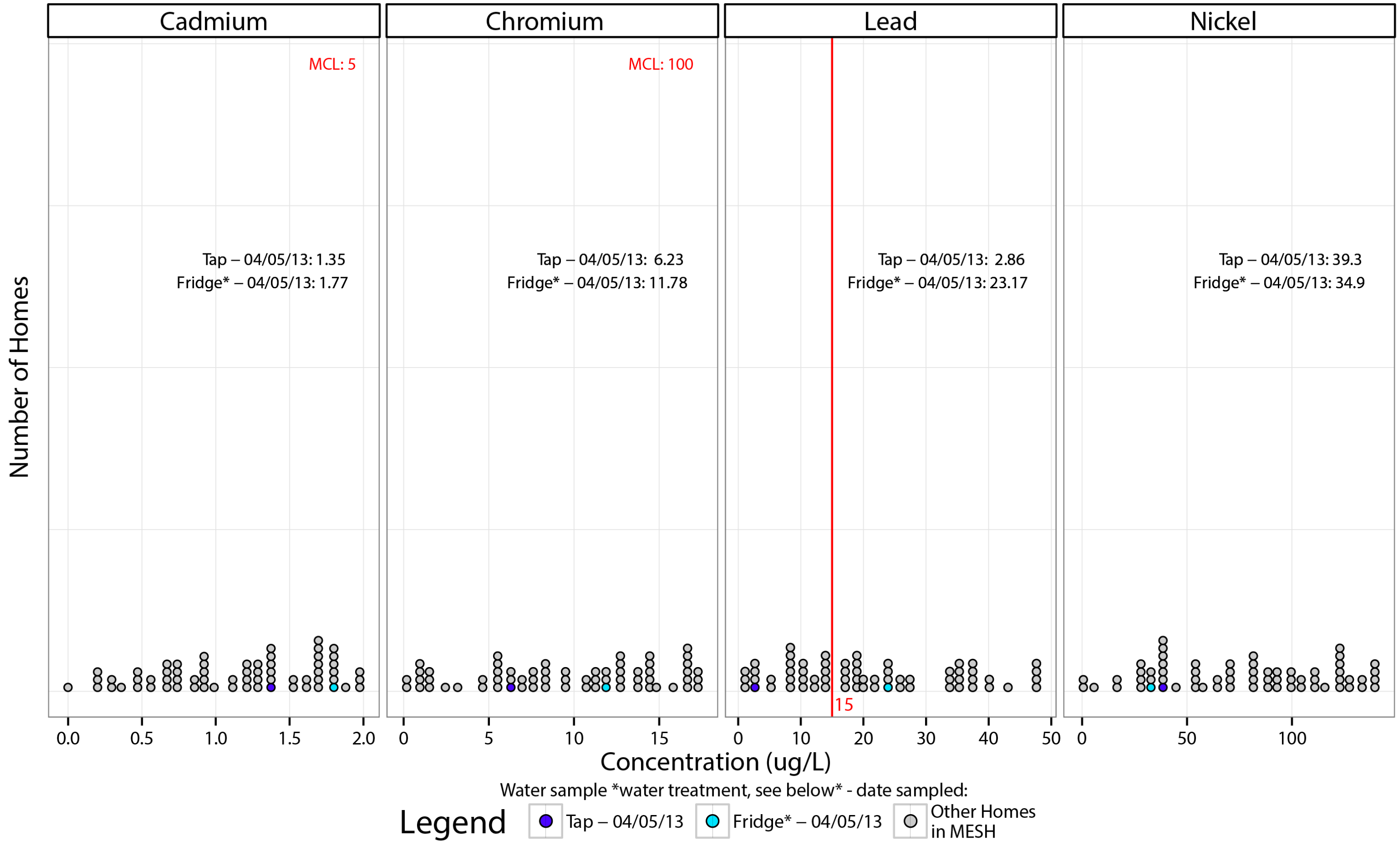

ND (None Detected): Metals with a level of "ND" have a concentration below what our laboratory can measure or detect.

Maximum Contaminant Level (MCL)

Water Treatments
* : Activated Carbon Filter
** : Reverse Osmosis
*** : Water Softener
**** : Cation Exchange Softener

Note: The dots represent all water samples from homes in MESH. Each dot represents one water sample from a home in MESH.

There is no MCL value for nickel.

Results References

Please visit these websites listed by subject to find information used in this results packet:

- **Arizona Department of Environmental Quality Soil Remediation Level**
  - The Arizona Department of Environmental Quality Soil Remediation Level (AZ SRL) is a residential yard soil screening level based on a health risk assessment not specific to conditions in Dewey-Humboldt. If a metal concentration in residential yard soil is above the AZ SRL, it suggests further study, but does not necessarily require cleanup. This level is set by the Arizona Department of Environmental Quality.
  - Please visit www.azsos.gov/public_services/title_18/18-07.htm and find the table underneath the section R18-7-210 - Notice of Remediation and Repository. Values are in the "Residential (mg/kg)" column.
- **Maximum Contaminant Level**
  - The Maximum Contaminant Level is the maximum amount of a contaminant allowed in drinking water so that it is still safe to use over the long-term. This level is set by the US Environmental Protection Agency.
  - Please visit water.epa.gov/drink/contaminants/index.cfm and scroll down to the "Inorganic Chemicals" heading. Values are in the "MCL or TT" column.
- **NHANES 50th Percentile** and **NHANES 95th Percentile**
  - The National Health and Nutrition Examination Survey (NHANES) is a study that periodically assesses the environmental exposures, health, and nutritional status of adults and children in the United States using a wide range of measures. The NHANES 50th Percentile level indicates that fifty percent (50%) of all samples are less than this concentration level, and fifty percent (50%) of all samples are greater than this concentration level. For the NHANES 95th Percentile, ninety-five percent (95%) of all samples are less than this concentration level, and only five percent (5%) are great than this concentration.
  - Please visit www.cdc.gov/exposurereport/pdf/FourthReport_UpdatedTables_Mar2013.pdf and browse to the "Metals and Metalloids" section. Find charts that read "Urinary *Metal*" in the top left corner. For example, "Urinary Total Arsenic." In the first column, find the "Age Group – 6-11 years;" this relates to the ages of children sampled. Next, move to the second column and find the row "09-10" for "6-11 years;" this shows the 2009-2010 results for children 6-11 years old. See values in this row that fall in the "50th" and "95th" percentiles columns.

Additional Information

Please visit these websites listed by subject to find additional information.

- **Agency for Toxic Substances and Disease Registry Toxicological Profiles**
  - The Agency for Toxic Substances and Disease Registry produces toxicological profiles, which summarize important studies on a wide range of contaminants, including metals.
  - www.atsdr.cdc.gov/toxprofiles/index.asp
- **American Academy of Pediatrics – Healthy Children**
  - Healthy Children is a website for parents that provides scientifically sound resources on child health and care which apply to infants through young adults. It is run by the American Academy of Pediatrics, a professional organization of 62,000 pediatricians.
  - www.healthychildren.org
- **Arizona Department of Environmental Quality**
  - The Arizona Department of Environmental Quality's (ADEQ) mission is to protect and enhance public health and the environment. ADEQ core responsibilities include pollution control, environmental monitoring and assessment, compliance management, cleanups, outreach and assistance, and policy development.
  - www.azdeq.gov
- **Arizona Department of Health Services**
  - The Arizona Department of Health Services (ADHS) promotes and protects the health of Arizona's children and adults. ADHS operates programs in behavioral health, disease prevention and control, health promotion, community public health, environmental health, maternal and child health, emergency preparedness and regulation of childcare and assisted living centers, nursing homes, hospitals, other health care providers and emergency services.
  - www.azdhs.gov
- **Clinical Laboratory Improvement Amendments** (referenced on page 3)
  - The Clinical Laboratory Improvement Amendments program is designed to ensure quality laboratory testing practices.
  - www.cms.gov/Regulations-and-Guidance/Legislation/CLIA/index.html
- **US Food and Drug Administration – Arsenic in Foods** (referenced on page 6)
  - The US Food and Drug Administration (FDA) is responsible for the safety and security of the nation's food supply. The FDA has been measuring arsenic in foods, including drinks, since 1991, with special emphasis on foods that children

are likely to eat or drink. This resource provides basic information on arsenic levels in specific foods.
  - www.fda.gov/Food/FoodborneIllnessContaminants/Metals/ucm280202.htm
- **National Health and Nutrition Examination Survey (NHANES)**
  - NHANES is a US Centers for Disease Control and Prevention program that annually examines about 5,000 people as young as 6 years of age across the United States to assess environmental exposures, health, and nutrition status.
  - www.cdc.gov/exposurereport/pdf/FourthReport.pdf
- **US Environmental Protection Agency**
  - The US Environmental Protection Agency (EPA)'s mission is to protect human health and the environment by developing and enforcing regulations, giving grants, studying environmental issues, and educating people about the environment.
  - www.epa.gov
- **US Environmental Protection Agency – Iron King Mine and Humboldt Smelter Superfund Site** (referenced on page 4)
  - The EPA maintains a website dedicated to reports and other resources concerning the Iron King Mine and Humboldt Smelter Superfund Site. In addition, some of this material, including the 2010 Iron King Mine and Humboldt Smelter Superfund Site Remediation Investigation, is available in print at the Dewey-Humboldt Town Library, located at 2735 S. Corral St., Humboldt, AZ.
  - http://yosemite.epa.gov/r9/sfund/r9sfdocw.nsf/ViewByEPAID/AZ0000309013

How to Reduce Your Exposure to Arsenic and Lead in Dewey-Humboldt, Arizona

Arizona Department of Health Services

ATSDR AGENCY FOR TOXIC SUBSTANCES AND DISEASE REGISTRY

This factsheet gives tips for protecting yourself and your family from coming into contact with arsenic and lead.

Why is exposure to arsenic and lead a concern in the Dewey-Humboldt area?

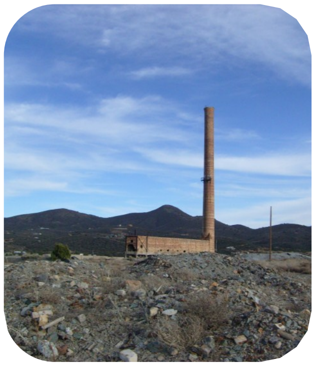

The Dewey-Humboldt area in Arizona has arsenic and lead from naturally occurring sources and from past mining and smelting activities. There is arsenic and lead in soil, dust and groundwater. Some foods can also contain arsenic.

Arsenic exposure at high doses can cause skin problems, stomach ache and nausea. Arsenic exposure over many years also raises the risk of bladder, lung, liver, and skin cancer. You can read more about the health effects of arsenic at: www.atsdr.cdc.gov/tfacts2pdf

Lead exposure at high doses can cause anemia, stomach ache, muscle weakness and damage to the brain and kidneys. In children, even low doses can affect IQ, ability to pay attention, and academic success, and cause behavioral problems. You can read more about the health effects of lead at: www.atsdr.cdc.gov/facts13.pdf

How can I reduce my exposures to arsenic and lead?

Make sure your drinking water is safe

In some places, groundwater in the Dewey-Humboldt area contains arsenic and other contaminants at levels above federal and state drinking water standards.

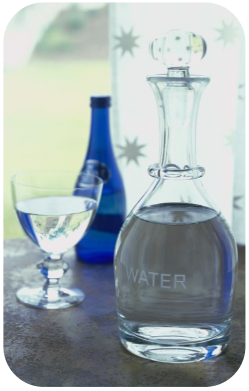

If you have a private well that you use for drinking water, you should test it for arsenic and lead. ADHS can help you with testing, see the table below.

- ✓ If a first test of your well water shows arsenic above 10 parts per billion (ppb), or lead 15 ppb or higher, collect and test a second sample before making any decisions about water treatment.
- ✓ If a second test shows that your well has arsenic above 10 ppb, or lead 15 ppb or higher, you should install a water treatment system that removes arsenic and lead.
  - * Use a different water source for drinking and cooking until you are able to install a water treatment system.
- ✓ You may need to test your well for metals every three years. In addition, ADHS recommends you test your well water yearly for bacteria.

If you get your water from the Humboldt Water System, your water is already tested for arsenic, lead and other contaminants.

The Humboldt Water System is a public drinking water system and is required to provide water that meets all state and federal drinking water standards. You can get a copy of the Humboldt Water System "Annual Consumer Confidence Report" by calling the Humboldt Water System at (928) 301-3723.

Help on groundwater wells, water treatment systems, and public water

For questions about …	Call:
… how often to test your private groundwater well	ADHS at (602) 364-3128
… which contaminants to test your well for	ADHS at (602) 364-3128
… how to find a water testing lab	ADHS lab at (602) 364-0728
… understanding water test results	ADHS at (602) 364-3128
… how to pick a water treatment system	Dr. Janick Artiola at the University of Arizona at (520) 621-3516
… water from the Humboldt Water System	Humboldt Water System at (928) 301-3723 or ADEQ at (602) 771-4641

Practice safe gardening

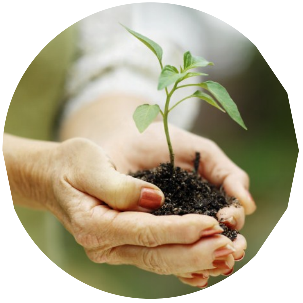

- ✓ Wash all fruits and vegetables whether they are homegrown or bought.
- ✓ Wash your hands after gardening and before eating or drinking.
- ✓ Limit the time children spend digging or playing in soil. A sandbox with clean sand is a healthier place for digging and playing.
- ✓ Research in Dewey-Humboldt found that lettuce, radishes, broccoli, Brussels sprouts, kale, cabbage, spinach, Swiss chard, and beets store more arsenic than other garden vegetables. Consider eating a limited amount of these vegetables from your garden.
- ✓ More information on gardening in soil possibly contaminated with arsenic is available from the University of Arizona Superfund Research Program at www.superfund.pharmacy.arizona.edu/content/gardenroots on the “Recommendations/Resources” tab.

Reduce dust in your home

- ✓ Limit the amount of soil you bring into your home by taking off coats, outerwear and shoes when entering your home and use a walk off mat at doorways to the outside.
- ✓ Vacuum carpets and rugs before mopping non-carpeted areas. If possible, use a vacuum with a high-efficiency particulate air (HEPA) filter.
- ✓ Use a wet mop on hard floors and clean window sills with wet rags regularly.
- ✓ Wash your hands and your children’s hands often, especially before eating and after outdoor activities.
- ✓ Be aware of other possible lead sources, like lead paint in older homes.

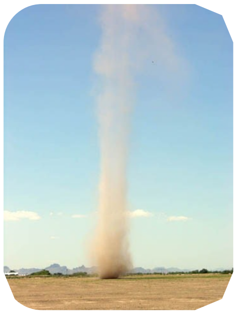

Ask your doctor to test your children’s blood for lead

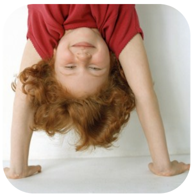

- ✓ At a minimum, all children in Yavapai County should have their blood tested for lead at age 1, and again at age 2. If you think a child of any age has come in contact with lead, then test that child’s blood for lead.
- ✓ If you suspect your children may be exposed lead, call your doctor or ADHS at (602) 364-4676 for more information.

Is there a medical test to show if I’ve been exposed to arsenic?

Testing urine for arsenic can show if you have been exposed in the last few days. But results cannot predict whether the arsenic levels in your body will affect your health. To learn more about testing urine for arsenic contact ATSDR at (415) 947-4316.

Who is working in my community?

The groups listed below are working on environmental cleanup, and looking into whether there are levels of arsenic and lead that could cause health effects to people in the community.

Agency		Contact Information
ADEQ	Arizona Department of Environmental Quality	(800) 234-5677 ext.7714641 ● azdeq.gov
ADHS	Arizona Department of Health Services	(602) 364-3128 ● azhealth.gov
ATSDR	Agency for Toxic Substances and Disease Registry	(415) 947-4316 ● atsdr.cdc.gov
EPA	Environmental Protection Agency	(415) 972-3068 ● epa.gov/region09/ironkingmine
UA	University of Arizona	(520) 307-3452 ● superfund.pharmacy.arizona.edu

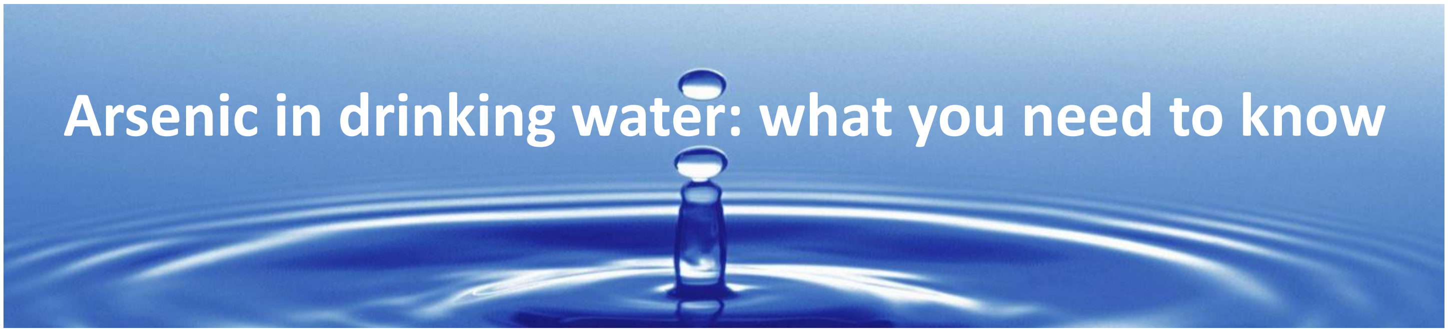

Arsenic in drinking water: what you need to know

Why should I be concerned about arsenic in my drinking water?

In Arizona, groundwater can contain arsenic that comes from natural sources, such as rocks and soils, and from human activities such as mining. Short-term exposure to very high doses of arsenic can cause poisoning. Long-term exposure to small doses has been linked to skin changes, cardiovascular effects, and cancer. Whether you are affected depends on how much arsenic you are exposed to and for how long, as well as your sensitivity to arsenic.

What level of arsenic in my drinking water is considered safe?

The United States Environmental Protection Agency has set the arsenic standard for drinking water at **10 parts per billion (ppb)** to protect consumers served by public water systems from the effects of long-term, chronic exposure to arsenic. 10 ppb is roughly equal to 5 teaspoons of ink in an Olympic-sized swimming pool. Read below to learn more about testing and treating your water to reach 10 ppb arsenic or less.

> Arsenic is colorless and tasteless, so **you cannot know it is there unless you test for it.**

How can I learn what level of arsenic is in my drinking water?

- *If you receive water from a* ***municipal or privately-owned water company***, they are required to test your water for arsenic. You should receive an annual water quality report from your water supplier by July 1st of each year.
- *If you drink* ***bottled water,*** bottled water companies are not required to report results of any water quality testing. If you choose bottled water, contact the supplier for information about impurities, including arsenic.
- *If you have* ***your own well***, you are responsible for testing and maintaining its water quality. Read below for more information on treatment systems.
  - To find a **State-certified testing laboratory**, contact the AZ Dept. of Health Services (ADHS) at **(602) 364-0720** or see this University of Arizona list: **cals.arizona.edu/pubs/garden/az1111.pdf**.
  - For help **understanding water test results**, contact Jennifer Botsford at ADHS at (602) 364-3128.

What can I do if there is arsenic in my water?

- *If arsenic in your water is* ***less than 10 ppb***, it is OK for drinking and cooking.
- *If arsenic in your water is between* ***10 – 200 ppb***, it is not an emergency, but make plans to get water for drinking and cooking from a different source, or install a home treatment system.
- *If arsenic in your water is* ***over 200 ppb***, DO NOT use this water for drinking and cooking. Use another source immediately and consider installing and maintaining a home treatment system. If the arsenic is less than 500 ppb, you can continue to use this water for bathing and washing.
- **Other sources of water** include bottled water or connecting to a public water supply. If you choose bottled water, contact the supplier for information about impurities, including arsenic, and be sure to clean and disinfect your water containers regularly.
- **Home treatment options** depend on factors such as how much arsenic you have, what other contaminants or conditions are present, and how much water you use.
  - MANY WATER TREATMENT TECHNIQUES DO NOT REMOVE ARSENIC, including particle filters, activated carbon filters (e.g. Brita), chlorine (bleach) disinfection, or heating/boiling your water. Water softening systems remove calcium and other minerals from hard water but DO NOT remove arsenic.

How do I choose a home water treatment system that removes arsenic?

There are many factors to consider when choosing a home water treatment system. For help with analyzing your water treatment needs and identifying an appropriate solution, **find a Water Professional** at: **wqa.org/members.cfm?section=1**.

Choosing a **point-of-use** (at a single tap) or **point-of-entry** (whole house) system will depend on how much water you need. Point-of-use treatment can often treat enough water needed for drinking and cooking.

It is important to:

- Test your water before choosing a system to find out your starting level of arsenic.
- Keep in mind that many systems remove a percentage of arsenic, but not all of it.
- Consider other contaminants and water conditions when choosing a system(s).
- Test your water after installing a system, to make sure it is effective at removing arsenic to 10 ppb or less.

- **Reverse osmosis (RO)** filters out contaminants by using pressure to force water through a special membrane. It is usually applied as a point-of-use system. **RO is very effective at removing arsenic** (removing up to 95%) and can reduce other contaminants as well.
  - Keep in mind that RO systems will increase your total water use and may need to be combined with other systems such as water softeners. RO systems require regular maintenance but do not require the addition of chemicals. There are a wide variety of choices for RO commercially, and many plumbers are familiar with it. Initial costs for installing an RO system are about $300 and up; annual costs are about $50 and up for membrane and filter replacement.
- **Distillation** is another effective technique for removing arsenic and other contaminants from water. However, AZ Department of Health Services does not recommend using only distilled water for all your drinking water needs.
- **Specialty media such as iron filters** are another option that can be easy to install as point-of-use or point-of-entry systems. However, these filters are relatively new to the home treatment market, so they may be harder to find and are more expensive, and their efficiency depends on other common water constituents.

Where can I buy a system?

You can purchase systems from:

- A plumber
- Home hardware stores
- The internet

Ensure you buy a certified system:

- NSF International: **nsf.org/certified/DWTU**
- The Water Quality Association: **wqa.org**

After installation:

Test your water to make sure treatment is effective. Maintain your system according to manufacturer's instructions.

Where can I learn more about arsenic in drinking water?

- U.S. Environmental Protection Agency: **water.epa.gov/lawsregs/rulesregs/sdwa/arsenic/index.cfm**
- Centers for Disease Control and Prevention: **cdc.gov/healthywater/drinking/private/wells/disease/arsenic.html**
- University of Arizona Superfund Research Program (click "Water Booklets and Videos" for free downloads or "Order Form" to request a hard copy): **http://www.superfund.pharmacy.arizona.edu/content/informational-materials**
- University of Arizona Cooperative Extension: **http://extension.arizona.edu//private-wells-publications**

Local contacts:

Janick Artiola, University of Arizona (Tucson)
Associate Professor & Water Quality Specialist
(520) 621-3516 / jartiola@cals.arizona.edu

Edessa Carr, Yavapai County Cooperative Extension (Prescott)
Water Resource Education Coordinator
(928) 445-6590 x227 / edessa@cals.arizona.edu

Superfund Research Program
The University of Arizona

superfund.pharmacy.arizona.edu

Authors: Sarah Wilkinson, PhD and Nathan Lothrop, MS
Expert reviewer: Janick Artiola, PhD
December, 2013
